# PCSK9 inhibition ameliorates microplastic-induced endothelial redox imbalance via SIRT6 modulation

**DOI:** 10.1186/s11658-025-00838-z

**Published:** 2025-12-22

**Authors:** Nunzia D’Onofrio, Isabella Donisi, Vitale Del Vecchio, Francesco Prattichizzo, Valeria Pellegrini, Michelangela Barbieri, Antonio Ceriello, Raffaele Marfella, Giuseppe Paolisso, Maria Luisa Balestrieri

**Affiliations:** 1 Department of Precision Medicine, University of Campania Luigi Vanvitelli, Via L. De Crecchio 7, 80138 Naples, Italy; 2 Department of Experimental Medicine, University of Campania Luigi Vanvitelli, Via Luciano Armanni 5, 80138 Naples, Italy; 3 IRCCS MultiMedica, Via Fantoli 16/15, 20138 Milan, Italy; 4 Department of Advanced Clinical and Surgical Sciences, University of Campania Luigi Vanvitelli, Piazza Miraglia, 80138 Naples, Italy; 5 Research Center for Environmental Pollution and Cardiovascular Diseases, University of Campania Luigi Vanvitelli, Piazza Miraglia, 80138 Naples, Italy

**Keywords:** Microplastics, PCSK9 inhibition, Endothelial dysfunction, SIRT6, Inflammation

## Abstract

**Background:**

Microplastics (MPs) have emerged as significant environmental pollutants, posing a threat to ecosystems and humans. The presence of MPs in atherosclerotic plaques, exacerbating cardiovascular risk, has been recently reported. However, the molecular mechanism underlying the effects of MPs on the vascular endothelium are still undefined. In this regard, this study aims to investigate the effects of MPs on endothelial cell function and redox state and the underlying mechanisms.

**Methods:**

Immortalized human aortic endothelial cells (teloHAEC), human umbilical vein endothelial cells (HUVEC), and human coronary artery endothelial cells (HCAEC) were treated with MPs in the form of polyethylene (PE) and polyvinyl chloride (PVC) alone (70 µg/mL) or combined PE (30 µg/mL) + PVC (30 µg/mL) (PE + PVC) for up to 48 h. The effects of MPs on cell viability were evaluated using CCK-8, and its role in endothelial function was evaluated by flow cytometric analyses, enzyme-linked immunosorbent assays (ELISA), and XF HS Seahorse bioanalyzer. Proprotein convertase subtilisin-kexin type 9 (PCSK9) levels were detected by reverse-transcription quantitative polymerase chain reaction (RT-qPCR) and immunoblotting. Molecular involvement of sirtuin 6 (SIRT6) was investigated through gene silencing.

**Results:**

Our study demonstrated that PE and PVC, alone or in combination, upregulated inflammatory mediators monocyte chemoattractant protein-1 (MCP-1), vascular cell adhesion molecule-1 (VCAM1), and intercellular adhesion molecule-1 (ICAM1) (*p* < 0.001), modulated the expression of autophagy markers anti-autophagy related 5 (ATG5) and p62, impaired mitochondrial metabolism by reducing maximal and basal respiration and adenosine triphosphate (ATP) production (*p* < 0.001), promoted reactive oxygen species (ROS) accumulation (*p* < 0.001) and cell cycle perturbations (*p* < 0.01), and increased apoptosis cell death (*p* < 0.001). These events were accompanied by a downregulation of sirtuin 6 (SIRT6) expression (*p* < 0.01) and an upregulation of PCSK9, at protein and messenger RNA (mRNA) levels (*p* < 0.01). Treatment with the PCSK9 inhibitor (iPCSK9) evolocumab ameliorated MP-induced cellular redox state imbalance, mitochondrial metabolism alteration, and SIRT6 downregulated levels (*p* < 0.01). SIRT6 transient silencing experiments denied the beneficial effects of iPCSK9 treatment, indicating that the pleiotropic functions of iPCSK9 may occur, at least in part, via modulation of SIRT6 and Forkhead box O3 (FOXO3A) expression levels.

**Conclusions:**

Overall, the results indicate that PCSK9 inhibition via evolocumab exhibits substantial promise in the prevention of MP-induced endothelial dysfunction, suggesting the PCSK9–SIRT6 axis as a new promising pathway to target in preventive strategies for cardiovascular risk caused by plastic pollution.

**Supplementary Information:**

The online version contains supplementary material available at 10.1186/s11658-025-00838-z.

## Introduction

The rapid spread of plastic pollution has raised concerns about its potential effects on human health. Microplastics (MPs) are contaminants of primary or secondary origin within the size range from 1 μm to 5 mm [1]. Primary MPs are small particles intentionally manufactured and added to industrial products, whereas secondary MPs result from the fragmentation of larger plastic pieces through ultraviolet rays, water, wind, and biological processes [2, 3]. Given their ubiquitous presence in the environment, such as soil, air, and water, humans are continuously exposed to MPs through inhalation, skin contact, and ingestion [4, 5]. After exposure, these particles can enter the circulatory system and migrate to different organs, where they can accumulate and possibly exert harmful effects on human health, triggering oxidative stress, inflammation, alterations in biochemical and energy metabolism, reduction of cell proliferation, and carcinogenicity [6–8]. To date, MPs have been identified in multiple human locations, including the liver [9], lungs [10, 11], gastrointestinal tract [12], central nervous system [13], female and male reproductive system [14–16], placenta [17, 18], and blood [19, 20]. Recently, a landmark study correlated the presence of MPs in atherosclerotic plaques with higher cardiovascular risk, compared with non-MP-associated plaques [21]. In particular, patients with atheromas presenting MPs displayed a significant increase in inflammatory markers, including interleukin-18 (IL-18), interleukin-1β (IL-1β), interleukin-6 (IL-6), and tumor necrosis factor-α (TNF-α), increased levels of lymphocyte and macrophage infiltration markers CD3 and CD68, and a decrease in collagen content [21]. Additionally, the presence of MPs in the plaques was associated with an increased risk of myocardial infarction, stroke, or any-cause mortality [21]. Furthermore, a subsequent study reported that, compared with nonpathologic aorta, the levels of MPs were significantly higher in coronary and carotid arteries with atherosclerotic plaques, supporting a role of MPs in atherosclerosis development [22].

In the progression of cardiovascular diseases (CVD), including atherosclerosis, endothelial cells (EC) play a critical role by the fine regulation of vascular homeostasis [23]. At the vascular level, MPs entering the circulatory system can interact with EC [24]. It has been observed, by in vitro and in vivo studies, that polystyrene MPs (PS-MPs) are able to induce endothelial activation and a significant decrease in cell viability, accompanied by an increase in oxidative stress and apoptosis [25, 26]. PS-MPs caused vascular barrier disruption, vascular malformation, and autophagy [26, 27]. However, knowledge of the impact of MPs on EC is still limited. In addition, no data about the effects of MPs polyethylene (PE) and polyvinyl chloride (PVC), two of the most widely produced and utilized synthetic polymers in the world [1], on EC have been described.

Proprotein convertase subtilisin/kexin type 9 (PCSK9) inhibitors (iPCSK9), such as evolocumab, are a class of drugs designed to lower low-density lipoprotein (LDL) cholesterol levels in the blood and have been demonstrated to ameliorate vascular endothelial functions [28]. Previous data reported that treatment with iPCSK9 determined multiple beneficial effects by counteracting endothelial inflammation, autophagy, and oxidative stress induced by IL-6 via sirtuin 3 modulation [28]. Indeed, PCSK9 modulation seems to be indirectly implicated in cellular epigenetic mechanisms via regulation of sirtuins (SIRT) [29], class III protein deacylases involved in the regulation of cellular homeostasis [30]. The nuclear SIRT6 plays a crucial role in the protection against inflammation, oxidative stress, premature endothelial senescence, and atherosclerotic plaque development [31–33]. However, no studies have explored the role of SIRT6 in the context of iPCSK9 vascular effects.

In this scenario, this study aimed to investigate the biological effects and molecular mechanism(s) induced by PE and PVC on EC function and to evaluate the ability of PCSK9 inhibition with evolocumab to oppose the endothelial cell impairment induced by MPs.

## Methods

### Cell growth and treatment

Immortalized endothelial human aorta cells (teloHAEC, CRL-4052), human umbilical vein endothelial cells (HUVEC, PCS-100-010), and human coronary artery endothelial cells (HCAEC, PCS-100-020) were purchased from the American Type Culture Collection and grown as already described [34]. EC cell lines were treated for a maximum time of 72 h with increasing concentrations (5–70 µg/mL) of polyethylene (PE, 434272, Sigma-Aldrich, St. Louis, MO, USA) or polyvinyl chloride (PVC, 1548076, U.S. Pharmacopeia, Rockville, MD, USA) microplastics (MPs). For combined treatment, EC were treated with 30 µg/mL of PE and increasing concentrations (5–70 µg/mL) of PVC or with 30 µg/mL PVC and increasing concentrations (5–70 µg/mL) of PE for 48 h. MPs were dissolved in ultrapure water (ddH_2_O), stored at 4 °C, and sonicated for 10 min using an ultrasonic bath (616.1010.04, FALC) before each experiment.

Evolocumab, a proprotein convertase subtilisin/kexin type 9 inhibitor (iPCSK9), was provided by Amgen Europe B.V. and diluted in Hanks’ balanced salt solution (HBSS–10 mM 4-(2-hydroxyethyl)-1-piperazineethanesulfonic acid (HEPES)). EC were pretreated for 8 h with 100 µg/mL iPCSK9 before being exposed to PE, PVC, or combined PE + PVC. Untreated cells (control cells, Ctr) were grown in complete culture medium with the corresponding highest volume of HBSS–10 mM HEPES.

### Cell viability assay

Cell Counting Kit-8 (CCK-8, Donjindo Molecular Technologies, Inc., Rockville, MD, USA) was used to determine the viability of EC cell lines treated with MPs alone or in combination and in the absence or presence of iPCSK9. After treatment, cells were incubated at 37 °C with 10 μL CCK-8 solution for 4 h, absorbance was measured at 450 nm with a microplate reader (model 680, Bio-Rad, Hercules, CA, USA), and viability is expressed as percentage of control. The combination index (Ci) was calculated using CompuSyn 1.0 software (Paramus, NJ, USA).

### RT-qPCR

After treatment, endothelial cells were collected by trypsinization, washed twice with phosphate-buffered saline (PBS), and lysed directly in QIAzol lysis reagent (79306, Qiagen, Milan, Italy), according to the manufacturer’s protocol. Total RNA was isolated from cell lysates by using the miRNeasy Mini kit (217004, Qiagen; Milan, Italy). RT-qPCR analyses were used to quantify human PCSK9 mRNA expression, using glyceraldehyde 3-phosphate dehydrogenase (*GAPDH*) as housekeeping gene. After assessing RNA concentration and quality with a NanoDrop 2000c spectrophotometer (Thermo Fisher Scientific, Waltham, MA, USA), genomic DNA contamination was removed by addition of gDNA Wipeout Buffer (205311, Qiagen; Milan, Italy). Specifically, genomic DNA elimination was carried out at 42 °C for 2 min. Then, total RNA was converted to complementary DNA (cDNA) by using a QuantiTect reverse transcription kit (205311, Qiagen; Milan, Italy) and Gene AMP PCR System 9700 (Applied Biosystems, MA, USA). The RT phases were 15 min at 42 °C and 3 min at 95 °C. QuantiTect primer assays (249900, Qiagen, Milan, Italy) were used for PCSK9 (QT00005509) and GAPDH (QT00079247). Amplifications was performed with the QuantiTect SYBR Green PCR kit (204143, Qiagen, Milan, Italy) on a CFX96 Real-Time System C1000 Touch thermal cycler (Biorad, Milan, Italy). The thermal cycling profile consisted of an initial activation step at 95 °C for 15 min, followed by 40 cycles of denaturation at 94 °C for 15 s, annealing at 55 °C for 30 s, and extension at 72 °C for 30 s. Melt-curve analysis was included to verify the specificity of the products. Each reaction was run in triplicate, and gene expression was quantified using the 2^−ΔΔCt^ method.

### Inflammatory mediators

The levels of inflammatory mediators MCP-1 (RAF081R, BioVendor, Brno, Czech Republic), VCAM1 (EH0326, FineTest, Hubei, China), and ICAM1 (EH0161, FineTest, Hubei, China) were determined in cell culture supernatant, as previously reported [28]. The absorbance at 450 nm was measured with a microplate reader (model 680, Bio-Rad, Hercules, CA, USA).

### Autophagy evaluation

Autophagy was detected by an Autophagy Assay Kit (ab139484, Abcam, Cambridge, UK) following the manufacturer's indication. Briefly, treated EC were staining with 1 µM Green Reagent for 40 min in the dark. Flow cytometry evaluation was performed using a fluorescence-activated cell sorting (FACS) CANTO II (BD Biosciences, San José, CA, USA), and data were analyzed by FlowJo version 10 software (Williamson Way, Ashland, OR, USA). For each sample, at least 20,000 events were recorded. To further support flow cytometry data, the protein expression levels of the autophagy markers autophagy-related protein 5 (ATG5) and sequestosome-1 (p62) were also assessed by immunoblotting.

### Apoptotic cell death analysis

Apoptosis was analyzed using a fluorescein isothiocyanate (FITC) Annexin V apoptosis detection kit (556547, BD Pharmigen, Franklin Lakes, NJ, USA). After treatment, EC were collected by trypsinization and resuspended in binding buffer 1× containing 2 μL Annexin V-FITC and 2 μL propidium iodide (PI) (20 μg/mL). The cell population was analyzed using a FACS CANTO II flow cytometer (BD Biosciences, San Jose, CA, USA), identifying four distinct cell populations in quadrants: Q1, necrotic cells (Annexin V⁻/PI⁺); Q2, late apoptotic cells (Annexin V⁺/PI⁺); Q3, early apoptotic cells (Annexin V⁺/PI⁻); Q4, viable cells (Annexin V⁻/PI⁻). At least 10,000 events were recorded for each sample, and results were evaluated by FlowJo version 10 software (FlowJo LLC, Ashland, OR, USA).

### Cell cycle assessment

After treatment, EC were detached by trypsinization and stained with PI solution consisting of 50 μg/mL PI, 0.1% sodium citrate, 25 μg/mL RNase A, and 0.1% Triton in phosphate-buffered saline (PBS). DNA content was analyzed using a FACS CANTO II flow cytometer (BD Biosciences, San Jose, CA, USA) and FlowJo version 10 software (FlowJo LLC, Ashland, OR, USA). At least 10,000 events were recorded for each sample.

### Detection of oxidative stress

Mitochondrial superoxide levels were assessed by MitoSOX Red mitochondrial superoxide indicator (M36008, Invitrogen, Waltham, MA, USA), following the manufacturer’s protocols. After treatments, EC were stained with 2 μM fluorescent probes for 30 min at 37 °C. Menadione (50 μM) was used as positive control. Images were obtained using a EVOS M5000 fluorescence microscope (Thermo Scientific, Rockford, IL, USA), and after trypsinization, fluorescent signals were quantified using a FACS CANTO II flow cytometer (BD Biosciences, San Jose, CA, USA). Data were analyzed by FlowJo version 10 software (FlowJo LLC, Ashland, OR, USA).

### Mitochondrial respiration analysis

Mitochondrial respiration analysis was performed using Seahorse XF Cell Mito Stress Test Kit (103010-100, Agilent Technologies, Santa Clara, CA, USA) as already described [35]. EC were seeded in Seahorse assay microplates with 100 μL of culture medium at a density of 8,000 cells/well. Before measurement, cells were washed with XF DMEM Assay medium supplemented with 2 mM glutamine, 1 mM pyruvate, and 10 mM glucose and incubated at 37 °C without CO_2_ for 1 h. The oxygen consumption rate (OCR) was evaluated using a XF HS Seahorse bioanalyzer (Agilent Technologies, Santa Clara, CA, USA) using oligomycin (1.5 μM), carbonyl cyanide-4(trifluoromethoxy) phenylhydrazone (FCCP) (1 μM), and rotenone/antimycin A (0.5 μM).

### *SIRT6* gene silencing

To induce transient *SIRT6* gene silencing, teloHAEC were transfected with 30 nM SIRT6 small interfering RNA (siRNA) Oligos set (Human) (siSIRT6, 43811171, Applied Biological Materi-als, Inc. Richmond, BC, Canada) or with the negative control siRNA (NT−/−) in serum- and antibiotic-free medium, using RNAifectin (vechicle) as transfectant reagent (G073, Applied Biological Materials, Inc. Richmond, BC, Canada). After 8 h of incubation, complete medium was added to the cells for another 12 h before starting treatments.

### Western blot analysis

Total protein content was extracted from EC with radioimmunoprecipitation assay (RIPA) buffer, and the concentrations were detected using the Bio-Rad Protein Assay kit (Bio-Rad, Hercules, CA, USA). Equal amounts (40 μg) of protein were separated by sodium dodecyl sulfate–polyacrylamide gel electrophoresis (SDS-PAGE) and transferred to nitrocellulose membranes. Then, the membranes were blocked in 1× Tris-buffered saline (TBS) 1% casein blocker (1610782, Bio-Rad, Hercules, CA, USA) for 1 h at room temperature and incubated with primary antibodies against PCSK9 (1:1000, ab28770, Abcam, Cambridge, UK), sirtuin 6 (SIRT6, 1:1000, ab62739, Abcam, Cambridge, UK), p62 (1:500, sc-28359, Cell Signaling Technology, Danvers, MA, USA), ATG5 (1:500, E-AB-10814, Elabscience Biotechnology Inc., Houston, TX, USA), and Forkhead Box O3 (FOXO3A, 1:1000, Orb161062) overnight at 4 °C. The next day, the membranes were incubated with peroxidase-conjugated secondary antibodies anti-rabbit immunoglobulin G (IgG) or anti-mouse IgG for 1 h at room temperature. Actin (1:2000, ab179467, Abcam, Cambridge, UK) and α-tubulin (1:2000, E-AB-20036, Elabscience Biotechnology Inc., Houston, TX, USA) were used as loading controls in different western blot experiments, depending on the experimental setting. Immunocomplexes were revealed using the Excellent Chemiluminescent Substrate kit (E-IR-R301, Elabscience Biotechnology Inc., Houston, TX, USA), and images were revealed by a ChemiDoc Imaging System with Image Lab 6.0.1 software (Bio-Rad Laboratories, Milan, Italy). The density of each band was quantified by ImageJ software 1.52n (Wayne Rasband, National Institutes of Health, Bethesda, MD, USA), compared with the loading control signal and expressed in arbitrary units (AU).

### Statistical analysis

Statistical analysis was performed by analysis of variance (ANOVA) followed by Tukey’s post hoc test by using GraphPad Prism 9.1.2 (Software Inc., La Jolla, CA, USA). Normality of data distribution was verified by Shapiro–Wilk test. *p*-Value (*P*) < 0.05 was considered statistically significant.

## Results

### MPs promoted cell viability reduction and inflammation in EC

**Fig. 1:**
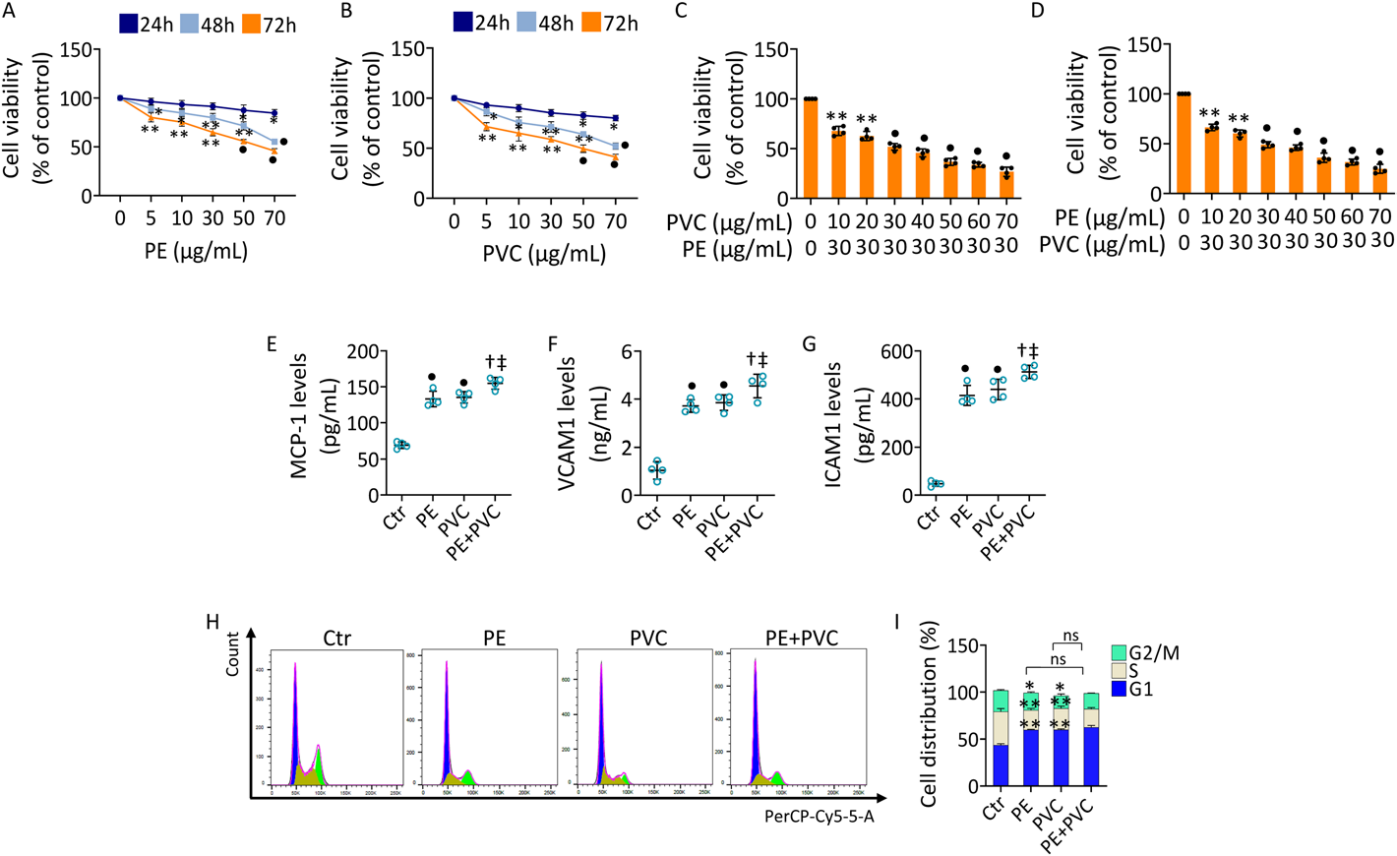
Effects of MPs on teloHAEC. Cell viability evaluated in teloHAEC exposed to different concentrations of **A** PE and **B** PVC (0–70 µg/mL) for 24, 48, and 72 h. **C**, **D** Cell viability assessed after 48 h of treatment with PE or PVC (30 µg/mL) and increasing concentration of PVC or PE (0–70 µg/mL), respectively. Evaluation of **E** MCP-1, **F** VCAM1, and **G** ICAM1 content in teloHAEC treated with PE (70 µg/mL), PVC (70 µg/mL), or PE + PVC (30 µg/mL + 30 µg/mL) for 48 h. **H**, **I** Representative cell cycle detection by FACS analysis in EC exposed to MPs. Data expressed as mean ± standard deviation (SD) of *n* = 4 independent experiments. *p < 0.05 versus 0 µg/mL or Ctr; **p < 0.01 versus 0 µg/mL or Ctr; •*p* < 0.001 versus 0 µg/mL or Ctr; †*p* < 0.05 versus PE; ‡*p* < 0.05 versus PVC; ns, not significant versus PE and PVC

To investigate the impact of MPs on endothelial redox dysfunction, teloHAEC, HUVEC, and HCAEC cell lines were treated with different concentrations of PE or PVC. Dose–response experiments in teloHAEC revealed that 24 h of treatment determined a reduction of cell viability of approximately 15% following exposure to 70 µg/mL of MPs (Fig. 1A, B).

After 48 h of incubation, an approximately 30% reduction in cell viability occurred with 30 µg/mL of PE or PVC (Fig. 1A, B). Similar results were observed in HUVEC (Supplementary Fig. S1) and HCAEC cells (Supplementary Fig. S2).

Exposure for 48 h to 50 µg/mL of PE or PVC impaired cell viability by about 35% (Fig. 1A, B), while a reduction of approximately 50% (*p* < 0.001 versus 0 µg/mL) was observed in TeloHAEC after treatment with 70 µg/mL of PE or PVC (Fig. 1A, B). Finally, 72 h of exposure to MPs resulted in a further decrease in cell viability, causing a reduction of approximately 42% with 30 µg/mL PE or PVC treatment and reaching IC_50_ with 50 µg/mL PE or PVC in teloHAEC (Fig. 1A, B). A similar trend was found in HUVEC (Supplementary Fig. S1) and HCAEC cells (Supplementary Fig. S2).

To explore the possible synergistic or additive effect of MPs, EC were exposed to PE and PVC in combination (PE + PVC). To this end, the concentration of PE and PVC (30 μg/mL), promoting a reduction of EC viability by around 30% after 48 h of treatment, was chosen to add increasing concentrations (up to 70 µg/mL) of PVC and PE, respectively.

Results revealed that, in teloHAEC, combined treatment for 48 h with PE (30 μg/mL) + PVC (30 μg/mL) reached a cell viability reduction of approximately 50% (*p* < 0. 001 versus 0 µg/mL), comparable to results obtained with PE or PVC 70 µg/mL alone (Fig. 1C, D). The resulting combination index (CI) was 0.60468 for PE + PVC and 0.56513 for PVC + PE, indicating a synergistic effect between MPs. Similar results were obtained in HUVEC and HCAEC, where the combined MPs (30 μg/mL + 30 μg/mL) determined a reduction in cell viability of about 50% (Supplementary Figs. S1C, D and S2C, D). Higher concentrations of PVC to PE resulted in excessive cell mortality, greatly exceeded the IC_50_, in all cell lines analyzed. On the basis of these results, subsequent experiments were performed on teloHAEC, HUVEC, and HCAEC using 70 µg/mL PE or PVC alone or combining 30 μg/mL of PE with 30 μg/mL of PVC.

Exposure to PE and PVC also induced an inflammatory state in teloHAEC, as evidenced by the increase of proinflammatory cytokine MCP-1 and adhesion molecules VCAM1 and ICAM1 levels (*p* < 0.001 versus Ctr). The increases in inflammatory molecules were also pronounced following the combined treatment (*p* < 0.05 versus PE, *p* < 0.05 versus PVC) (Fig. 1E–G). Likewise, MPs induced inflammatory state in HUVEC and HCAEC cell lines (Supplementary Figs. S1E–G and S2E–G).

Cell cycle distribution analysis in teloHAEC cells showed the ability of PE and PVC to induce cell cycle arrest in G1 phase (*p* < 0.01 versus Ctr) with a concomitant decrease in the S (*p* < 0.01 versus Ctr) and G2 population (*p* < 0.05 versus Ctr) after treatment with MPs (Fig. 1H, I). Similarly, incubation with MPs was able to induce cell cycle impairment also in HUVEC and HCAEC (Supplementary Figs. S1H, I and S2H, I).

### PE and PVC triggered autophagy and apoptotic cell death

**Fig. 2:**
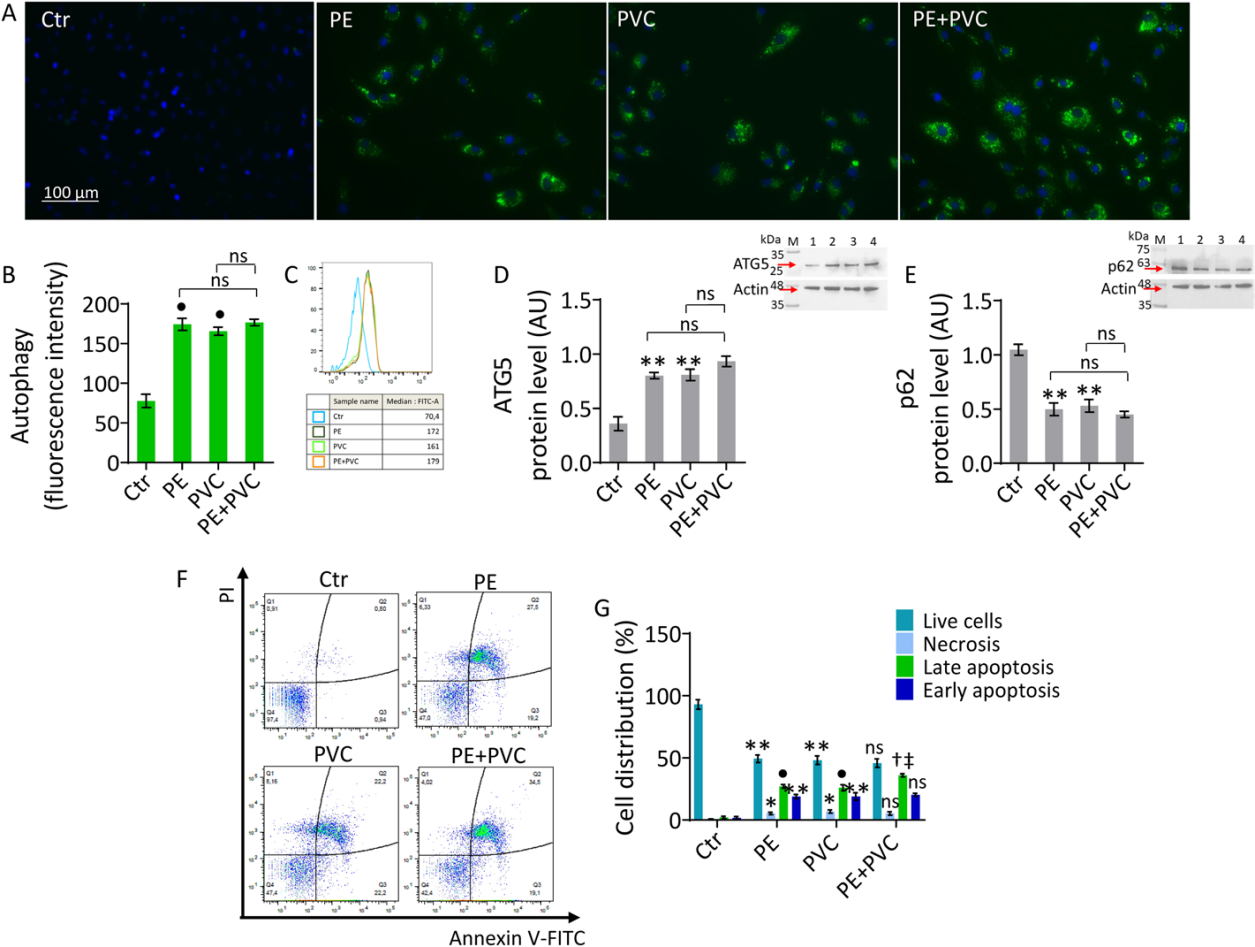
MPs triggered cell death mechanisms. **A**–**C** Representative fluorescent images and FACS analysis of autophagy. Immunoblotting analysis of **D** ATG5 and **E** p62 protein levels in teloHAEC treated with PE (70 µg/mL), PVC (70 µg/mL), or PE + PVC (30 µg/mL each one) for 48 h. Results expressed as median fluorescence intensity (MFI). **F**, **G** Representative annexin V-FITC and PI-staining detected by FACS analysis. Data expressed as mean ± SD of *n* = 3 experiments. Q1: necrotic cells; Q2: late apoptotic cells; Q3: early apoptotic cells; Q4: viable cells. M, molecular weight markers; lane 1, Ctr; lane 2, PE; lane 3, PVC; lane 4, PE + PVC. Scale bars = 100 μm. **p < 0.01 versus Ctr; •*p* < 0.001 versus Ctr; †*p* < 0.05 versus PE; ‡*p* < 0.05 versus PVC; ns, not significant versus PE and PVC

The increase in inflammation induced by MPs led us to investigate the autophagic process and apoptosis (Fig. 2 and Supplementary Fig. S3). In teloHAEC, exposure to MPs promoted autophagy (1.8-fold change, *p* < 0.001 versus Ctr), as confirmed by increased pro-autophagic ATG5 (*p* < 0.01 versus Ctr) and decreased anti-autophagic p62 (*p* < 0.01 versus Ctr) protein levels (Fig. 2A–E). These results were observed also in HUVEC and HCAEC cell lines (Supplementary Fig. S3A–C, F–H). Annexin/PI measurements revealed that PE and PVC caused teloHAEC cell death via apoptosis with a reduction of live cells (*p* < 0.01 versus Ctr) and an increase of late (*p* < 0.001 versus Ctr) and early apoptosis (*p* < 0.01 versus Ctr). Compared with single MPs, combined treatment (PE + PVC) aggravated the EC rate in late apoptosis (about 1.5-fold, *p* < 0.05 versus PE, *p* < 0.05 versus PVC). In addition, the results showed an increase in necrosis following MPs treatment (*p* < 0.05 versus Ctr) (Fig. 2F, G). In HUVEC, a similar decrease of live cells (*p* < 0.01 versus Ctr) with an increase of late (*p* < 0.01 versus Ctr) and early apoptosis (*p* < 0.001 versus Ctr) was observed. In addition, increased necrotic cells in PE + PVC were observed (*p* < 0.05 versus PE, *p* < 0.05 versus PVC) (Supplementary Fig. S3D,E). Finally, in HCAEC, exposure to PE or PVC caused a decrease in live cells (*p* < 0.01 versus Ctr), an increase in late (*p* < 0.001 versus Ctr) and early apoptosis (*p* < 0.05 versus Ctr), and necrosis (*p* < 0.05 versus Ctr) (Supplementary Fig. S3I, J).

### PE and PVC increased mitochondrial oxidative stress and impaired respiration

**Fig. 3:**
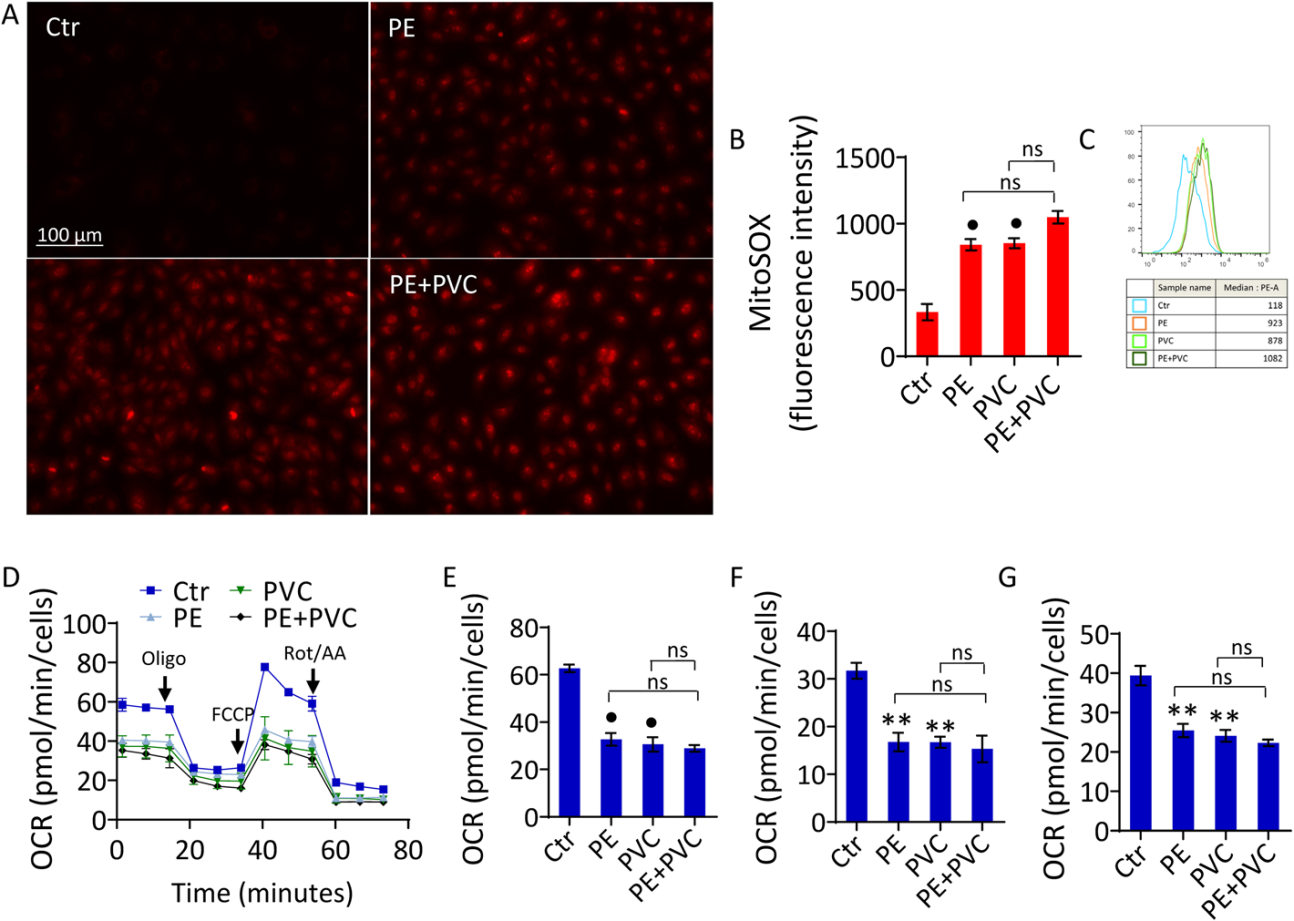
MPs induced mitochondrial impairment. **A**–**C** Representative fluorescent images and FACS analysis of mitochondrial ROS and Seahorse analysis of **D** oxygen consumption rate, **E** maximal respiration, **F** ATP production, and **G** basal respiration measured in teloHAEC stimulated with PE, PVC, and PE + PVC for 48 h. Results expressed as median fluorescence intensity (MFI). Data expressed as mean ± SD of *n* = 3 experiments. Scale bars = 100 μm. **p < 0.01 versus Ctr; •*p* < 0.001 versus Ctr; ns, not significant versus PE and PVC

Given the crucial role of mitochondrial reactive oxygen species (ROS) in the observed cellular processes [36], we explored the effects of PE and PVC on mitochondrial ROS levels. Results obtained from median fluorescence intensity (MFI) showed an extensive accrual of mitochondrial ROS during MP stimulation of teloHAEC, as evidenced by the increase in red fluorescence intensity (1.9-fold change) (*p* < 0.001 versus Ctr) correlated to ROS level generation (Fig. 3A–C). Moreover, treatment with MPs also induced alteration of mitochondrial respiration, showing a reduction of maximal respiration (*p* < 0.001 versus Ctr) associated with a decrease of ATP production (*p* < 0.01 versus Ctr) and basal respiration (*p* < 0.01 versus Ctr) (Fig. 3D–G).

In a similar way, PE and PVC provoked the generation of mitochondrial ROS (*p* < 0.001 versus Ctr) and mitochondrial respiration dysfunction, through a decrease of maximal respiration (*p* < 0.05 versus Ctr), ATP production (*p* < 0.05 versus Ctr), and basal respiration (*p* < 0.05 versus Ctr) in HUVEC and HCAEC cell lines (Supplementary Fig. S4).

### PE and PVC modulated SIRT6 and PCSK9 levels

**Fig. 4:**
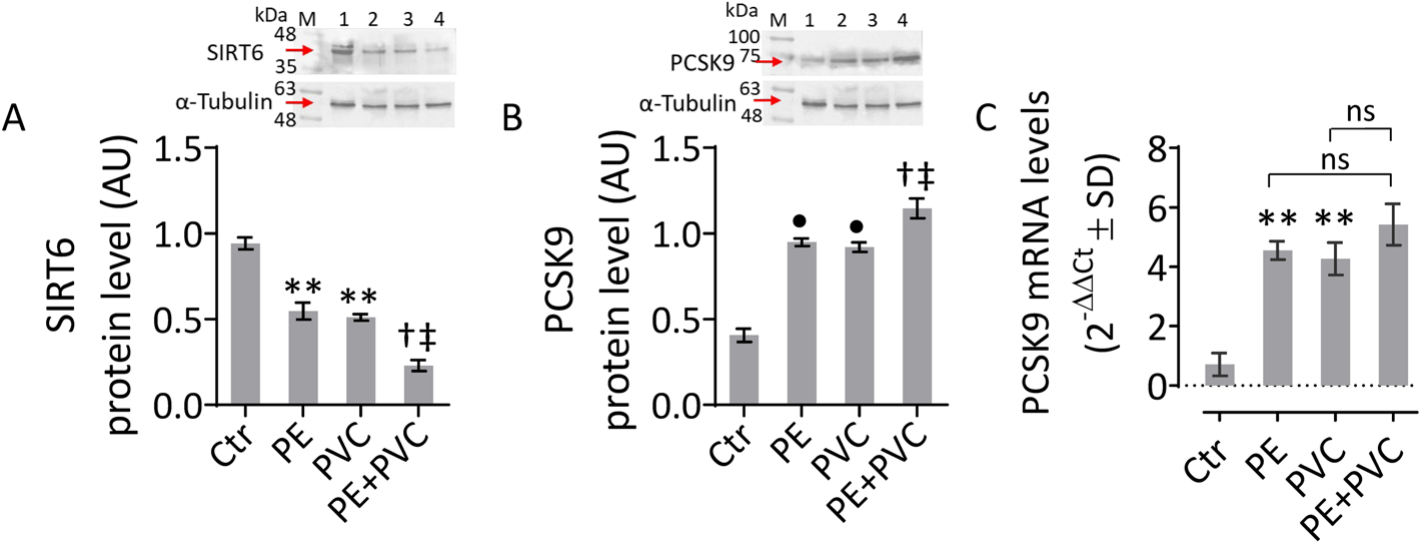
MP modulation of PSCK9 and SIRT6. **A** Immunoblotting analysis of SIRT6 protein levels. **B**, **C** PCSK9 protein and mRNA levels in teloHAEC treated with PE and PVC alone or in combination (PE + PVC). Data expressed as mean ± SD of *n* = 3 experiments. M, molecular weight markers; lane 1, Ctr; lane 2, PE; lane 3, PVC; lane 4, PE + PVC. **p < 0.01 versus Ctr; •*p* < 0.001 versus Ctr; †*p* < 0.05 versus PE; ‡*p* < 0.05 versus PVC; ns, not significant versus PE and PVC

The MP redox-induced imbalance resulted in the downregulation of SIRT6, a key regulator of endothelial function and redox homeostasis [31]. In detail, immunoblotting data revealed SIRT6 downregulation in PE- and PVC-treated cells (*p* < 0.01 versus Ctr) and in all endothelial cells stimulated with PE + PVC (*p* < 0.05 versus PE, *p* < 0.05 versus PVC) (Fig. 4A and Supplementary Fig. S5). Of interest, the SIRT6 downregulation was accompanied by modulation of PCSK9, a new crucial player in vascular disease progression [37]. Data showed that treatments with PE and PVC led to an overexpression of PCSK9 protein levels (*p* < 0.001 versus Ctr), as well as the combined treatment PE + PVC (*p* < 0.05 versus PE, *p* < 0.05 versus PVC) (Fig. 4), in all EC analyzed (Fig. 4B and Supplementary Fig. S5).

The response to MP stimulation was highly concordant among immortalized teloHAEC, primary HCAEC and HUVEC cells, confirming that teloHAEC represents a powerful cellular model to study endothelial cell dysfunction and the underlined molecular mechanism(s) [34]. In this regard, teloHAEC were used to assess the PCSK9 mRNA levels under MP stimulation. Results of RT-qPCR analysis confirmed the increase in PCSK9 levels following PE and PVC treatments in teloHAEC (*p* < 0.01 versus Ctr) (Fig. 4C).

### PCSK9 inhibition attenuated MP-related EC dysfunction

**Fig. 5:**
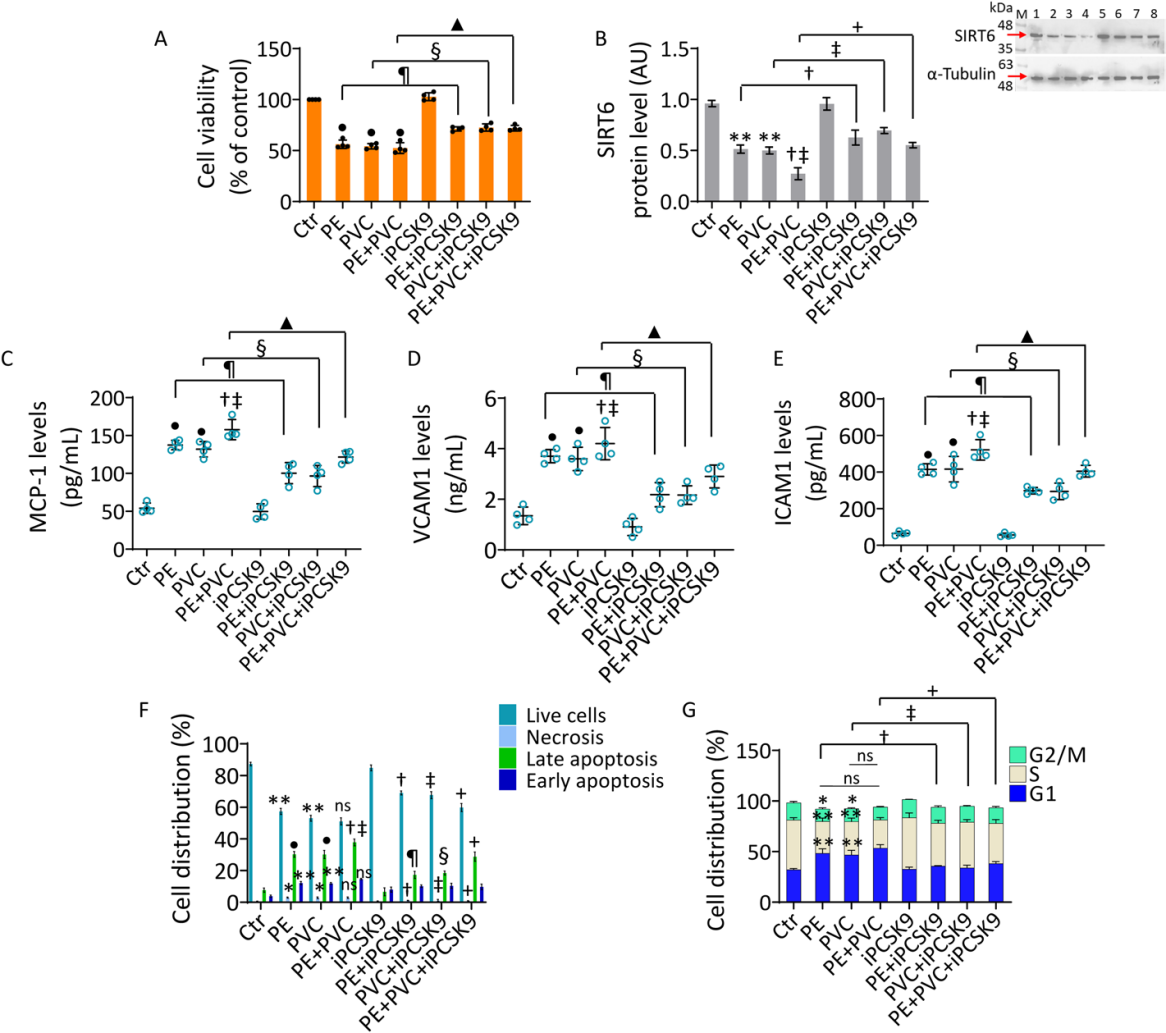
iPCSK9 opposed the MP-related inflammation. **A** TeloHAEC viability evaluated after treatment with PE and PVC alone or combined PE + PVC, or pretreated with iPCSK9 (100 ng/mL) for 8 h and then exposed to PE, PVC, and PE + PVC. **B** Immunoblotting analysis of SIRT6 protein levels and ELISA assays of **C** MCP-1, **D** VCAM1, and **E** ICAM1. **F** Representative annexin V-FITC and PI-staining detected by FACS analysis and **G** cell cycle investigation. Data expressed as mean ± SD of *n* = 4 experiments. M, molecular weight markers; lane 1, Ctr; lane 2, PE; lane 3, PVC; lane 4, PE + PVC; lane 5, iPCSK9; lane 6, PE + iPCSK9; lane 7, PVC + iPCSK9; lane 8, PE + PVC + iPCSK9. *p < 0.05 versus Ctr; **p < 0.01 versus Ctr; •*p* < 0.001 versus Ctr; †*p* < 0.05 versus PE; ‡*p* < 0.05 versus PVC; + *p* < 0.05 versus PE + PVC; ¶*p* < 0.01 versus PE; §*p* < 0.01 versus PVC; ▲*p* < 0.01 versus PE + PVC; ns, not significant versus PE and PVC

To evaluate the role of PCSK9 in EC treated with MPs, the PCSK9 inhibitor (iPCSK9), evolocumab, involved in decreasing vascular inflammatory risk [28], was investigated in teloHAEC cells. The results indicated that pretreatment with 100 µg/mL iPCSK9 for 8 h was effective in opposing MP-induced cytotoxicity and inflammatory cascade (Fig. 5). In detail, ELISA assays showed the ability of iPCSK9 to attenuate the increase of key pro-inflammatory modulators including MCP-1, VCAM1, and ICAM1 (*p* < 0.01 versus PE, *p* < 0.01 versus PVC, *p* < 0.01 versus PE + PVC) (Fig. 5C–E). Of note, iPCSK9 counteracted the MP-induced decrease in SIRT6 (*p* < 0.05 versus PE, *p* < 0.05 versus PVC, *p* < 0.05 versus PE + PVC), supporting the beneficial effects of evolocumab in EC during MP exposure (Fig. 5B).

Treatment with iPCSK9 also attenuated the apoptotic cell death, ameliorating the live cell number (*p* < 0.05 versus PE, *p* < 0.05 versus PVC, *p* < 0.05 versus PE + PVC) and decreasing late apoptosis (*p* < 0.01 versus PE, *p* < 0.01 versus PVC, *p* < 0.05 versus PE + PVC), and necrosis (*p* < 0.05 versus PE, p < 0.05 versus PVC, *p* < 0.05 versus PE + PVC) (Fig. 5F and Supplementary Fig. S5A). In addition, when cell cycle analysis was performed, data indicated that iPCSK9 opposed the negative effects of MPs, decreasing the EC population in G1 phase (*p* < 0.05 versus PE, *p* < 0.05 versus PVC, *p* < 0.05 versus PE + PVC) (Fig. 5G and S5B).

### iPCSK9 counteracted MP-induced autophagy and mitochondrial damage

**Fig. 6:**
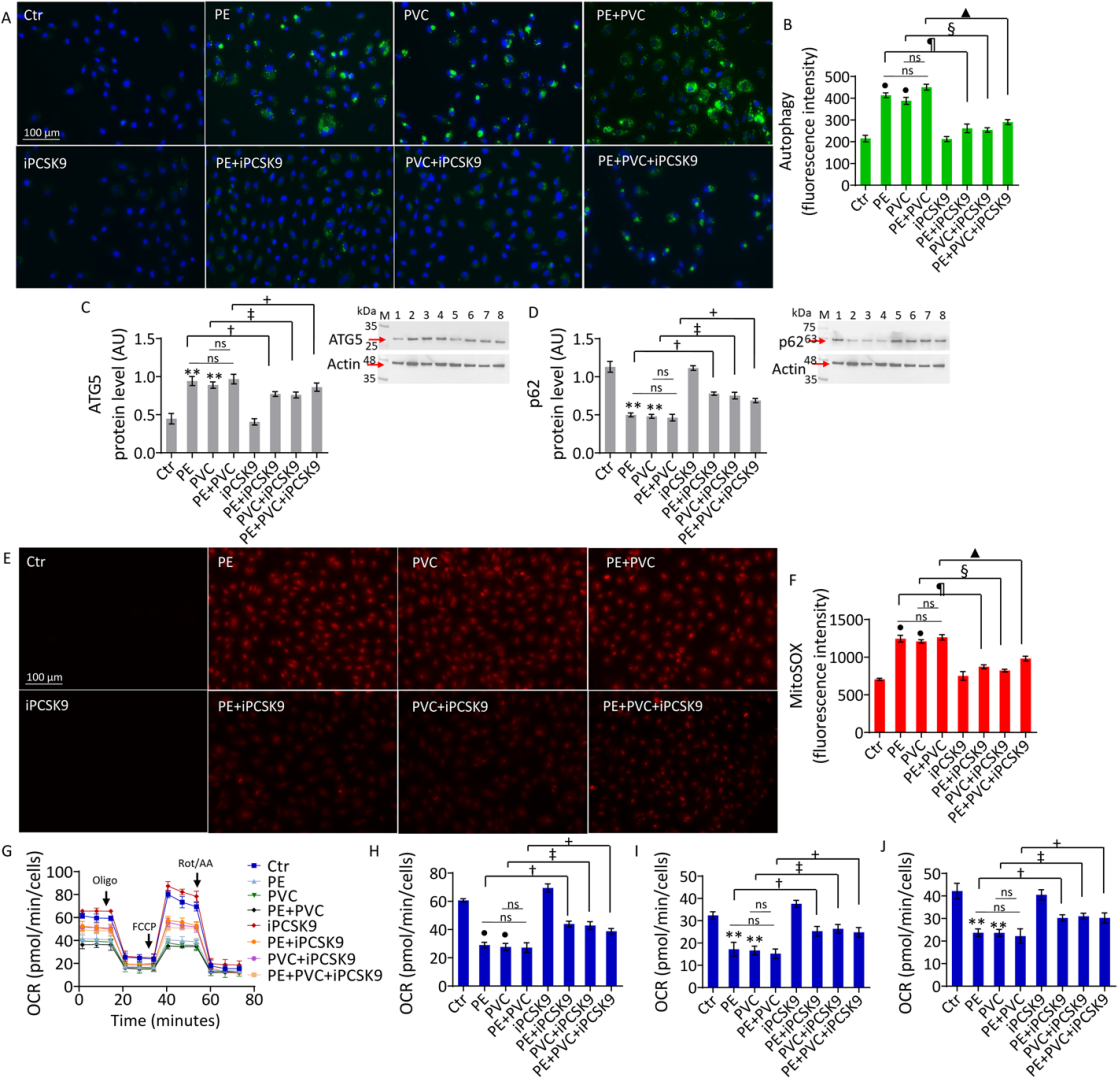
iPCSK9 counteracted MP-induced autophagy and mitochondrial alteration. **A**, **B** Representative images and FACS analysis graph of autophagy and immunoblotting analysis of **C** ATG5 and **D** p62. **E**, **F** Representative images and graph of mitochondrial ROS and **G** oxygen consumption rate, **H** maximal respiration, **I** ATP production, and **J** basal respiration measured with Seahorse analyzer in teloHAEC treated with PE and PVC alone or combined PE + PVC, or pretreated with iPCSK9 and then exposed to PE, PVC, and PE + PVC. Results expressed as median fluorescence intensity (MFI). Data expressed as mean ± SD of *n* = 3 experiments. M, molecular weight markers; lane 1, Ctr; lane 2, PE; lane 3, PVC; lane 4, PE + PVC; lane 5, iPCSK9; lane 6, PE + iPCSK9; lane 7, PVC + iPCSK9; lane 8, PE + PVC + iPCSK9. Scale bars = 100 μm. *p < 0.05 versus Ctr; **p < 0.01 versus Ctr; •*p* < 0.001 versus Ctr; †*p* < 0.05 versus PE; ‡*p* < 0.05 versus PVC; + *p* < 0.05 versus PE + PVC; ¶*p* < 0.01 versus PE; §*p* < 0.01 versus PVC; ▲*p* < 0.01 versus PE + PVC; ns, not significant versus PE and PVC

The induction of autophagy by MPs was opposed by iPCSK9 pretreatment (0.6-fold change) (*p* < 0.01 versus PE, *p* < 0.01 versus PVC, *p* < 0.01 versus PE + PVC), restoring ATG5 (*p* < 0.01 versus PE, *p* < 0.01 versus PVC, *p* < 0.01 versus PE + PVC) and p62 (*p* < 0.01 versus PE, *p* < 0.01 versus PVC, *p* < 0.01 versus PE + PVC) protein levels (Fig. 6A–D and Supplementary Fig. S5C). iPCSK9 treatment also counteracted the MP-induced mitochondrial ROS accumulation (0.7-fold change) (*p* < 0.01 versus PE, *p* < 0.01 versus PVC, *p* < 0.01 versus PE + PVC) and mitochondrial respiration impairment (Fig. 6E–J and Supplementary Fig. S5D). In particular, iPCSK9 ameliorated maximal respiration (*p* < 0.05 versus PE, *p* < 0.05 versus PVC, *p* < 0.05 versus PE + PVC), ATP production (*p* < 0.05 versus PE, *p* < 0.05 versus PVC, *p* < 0.05 versus PE + PVC), and basal respiration (*p* < 0.05 versus PE, *p* < 0.05 versus PVC, *p* < 0.05 versus PE + PVC) of EC treated with MPs (Fig. 6G–J).

### SIRT6 silencing denied the protective effects of iPCSK9

**Fig. 7:**
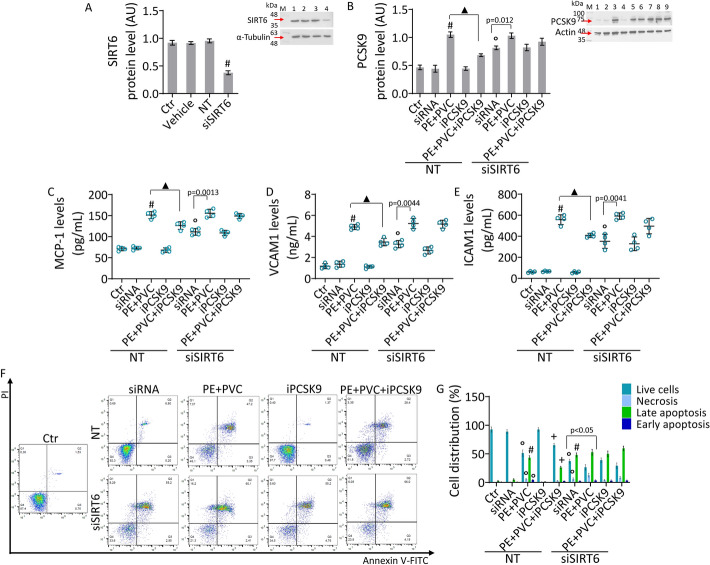
SIRT6 silencing opposed the effects of iPCSK9 on inflammation and apoptosis. **A** Immunoblotting analysis of SIRT6 protein levels in teloHAEC treated with the empty transfection reagent (Vehicle) or transfected with negative control siRNA (NT) or with SIRT6 siRNA (siSIRT6). M, molecular weight markers; lane 1, Ctr; lane 2, Vehicle; lane 3, NT; lane 4, siSIRT6. **B** Immunoblotting analysis of PCSK9 protein levels and ELISA assays of **C** MCP-1, **D** VCAM1 and **E** ICAM1. **F**, **G** Representative dot plots and FACS analysis of annexin V-FITC and PI-staining of EC transfected with NT or siSIRT6 and exposed to PE + PVC alone or pretreated with iPCSK9 (100 µg/mL) before MP stimulation. M, molecular weight markers; lane 1, Ctr; lane 2, NT; lane 3, NT + PE + PVC; lane 4, NT + iPCSK9; lane 5, NT + PE + PVC + iPCSK9; lane 6, siSIRT6; lane 7, siSIRT6 + PE + PVC; lane 8, siSIRT6 + iPCSK9; lane 9, siSIRT6 + PE + PVC + iPCSK9. Q1: necrotic cells; Q2: late apoptotic cells; Q3: early apoptotic cells; Q4: viable cells. Data are expressed as mean ± SD of *n* = 3 experiments. °*p* < 0.01 versus NT; #*p* < 0.001 versus NT; + *p* < 0.05 versus PE + PVC; ▲*p* < 0.01 versus PE + PVC

Given the data relative to SIRT6 modulation under iPCSK9 treatment and considering the role played by this protein in regulating inflammatory responses and oxidative stress at the vascular level [31], we explored whether the protective effects of iPCSK9 could occur via SIRT6 (Fig. 7). First, transient gene silencing experiment indicated that SIRT6-silenced EC (siSIRT6) showed increased PCSK9 expression levels (*p* < 0.001 versus NT) and counteracted the protective role of iPCSK9 against MP-increased PCSK9 levels. Moreover, siSIRT6 showed an increase in pro-inflammatory state, as evidenced by the increase in MCP-1, VCAM1, and ICAM1 (*p* < 0.01 versus NT) and opposed the beneficial effects of iPCSK9 in ameliorating MP-induced inflammation (Fig. 7B–E). siSIRT6 also caused apoptosis (*p* < 0.001 versus NT), worsened the effects of MPs on EC death (siSIRT6 + PE + PVC) (*p* < 0.01 versus PE + PVC), and counteracted the protective effects of iPCSK9 against MP-increased apoptosis, indicating the involvement of SIRT6 in iPCSK9 functions (Fig. 7F, G).

### SIRT6 silencing counteracted the beneficial effects of iPCSK9 on oxidative stress

**Fig. 8:**
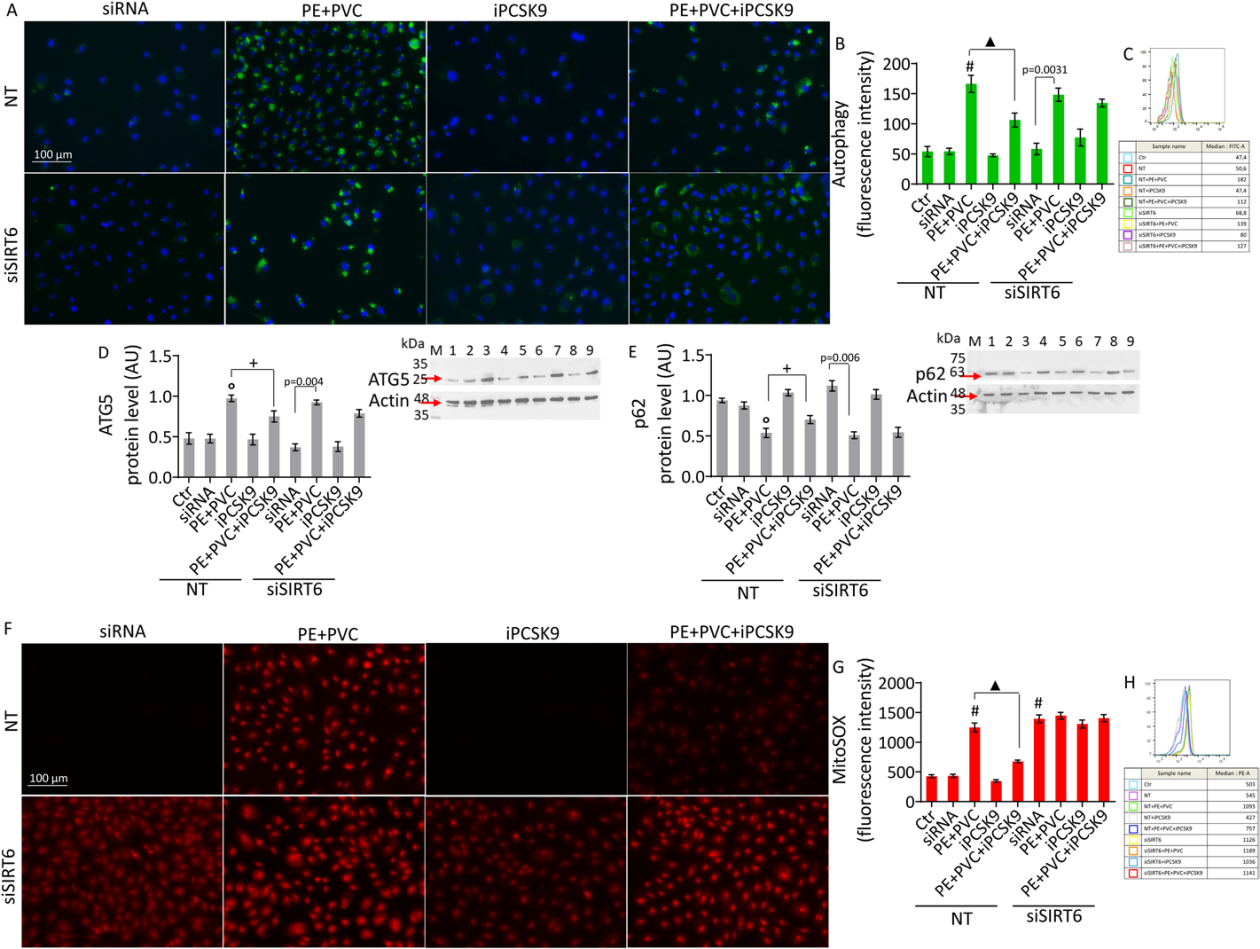
SIRT6 suppression denied the ability of iPCSK9 to counteract oxidative stress. **A**–**C** Representative images and FACS analysis of autophagy and immunoblotting analysis of **D** ATG5 and **E** p62 protein levels in teloHAEC transfected with negative control (NT) or with SIRT6 siRNA (siSIRT6) and then exposed to PE + PVC (NT or siSIRT6 + PE + PVC), to 100 µg/mL iPCSK9 (NT or siSIRT6 + iPCSK9), or pretreated with 100 µg/mL iPCSK9 for 8 h before exposure to combined MPs (NT or siSIRT6 + PE + PVC + iPCSK9). **F**–**H** Representative images by fluorescence microscopy and FACS analysis of mitochondrial ROS levels. Results expressed as median fluorescence intensity (MFI). Scale bars = 100 µm. M, molecular weight markers; lane 1, Ctr; lane 2, NT; lane 3, NT + PE + PVC; lane 4, NT + iPCSK9; lane 5, NT + PE + PVC + iPCSK9; lane 6, siSIRT6; lane 7, SIRT6 + PE + PVC; lane 8, siSIRT6 + iPCSK9; lane 9, SIRT6 + PE + PVC + iPCSK9. Data expressed as mean ± SD of *n* = 3 experiments. °*p* < 0.01 versus NT; #*p* < 0.001 versus NT; + *p* < 0.05 versus PE + PVC; ▲*p* < 0.01 versus PE + PVC

Compared with control cells, SIRT6-silenced EC showed higher levels of mitochondrial ROS (*p* < 0.001) as evidenced by the increase in red fluorescence intensity. In addition, transient silencing of SIRT6 inhibited the iPCSK9 capacity to counteract the accumulation of ROS induced by MPs (Fig. 8F–H). In contrast, silencing of SIRT6 in endothelial cells did not promote the autophagy process and did not affect MP-induced autophagy, but it opposed the effects of PCSK9 in inhibiting MP-mediated autophagy (Fig. 8A–E).

### FOXO3A involvement in MP-induced endothelial damage

**Fig. 9:**
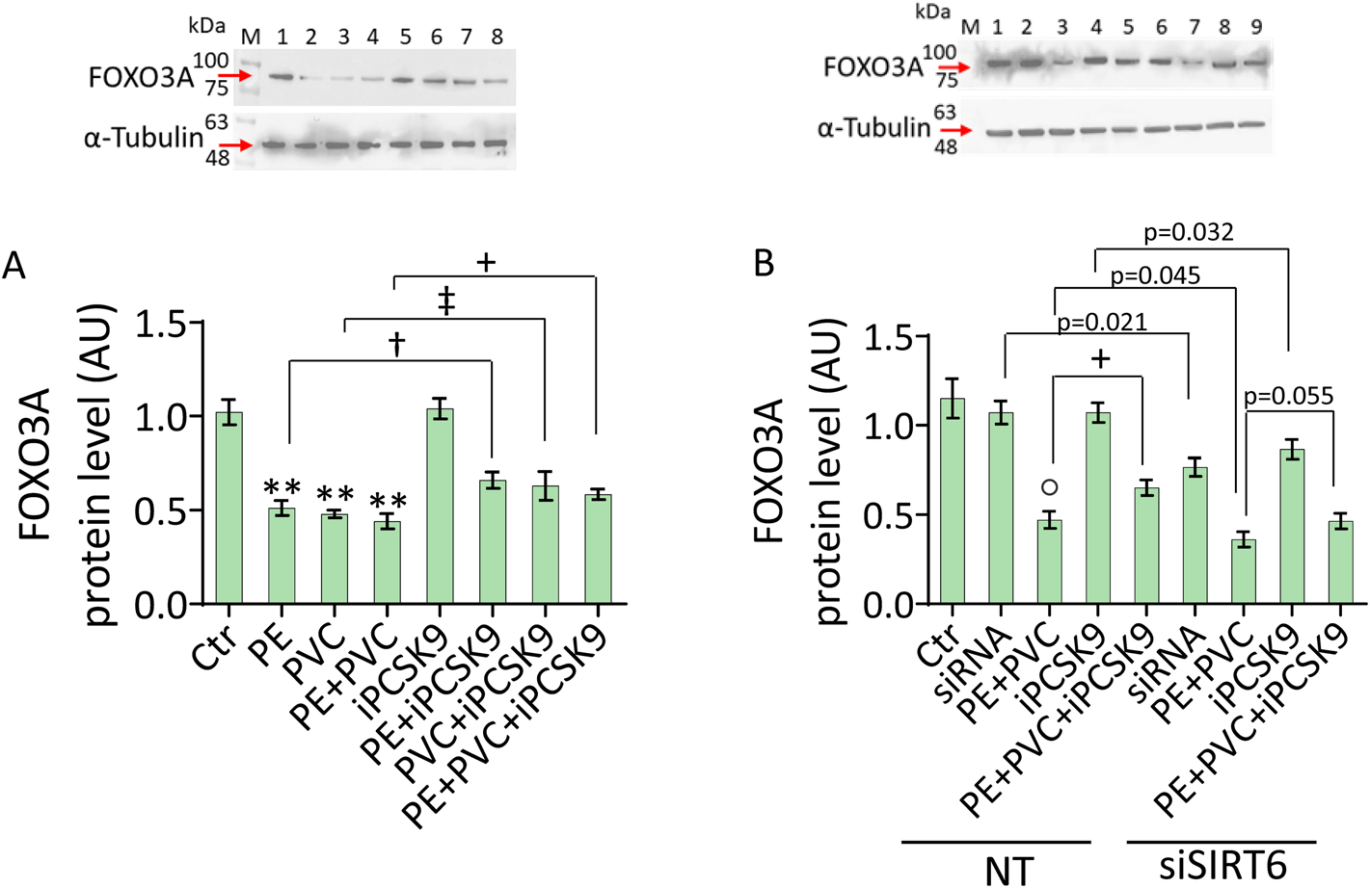
Involvement of FOXO3A. **A** Immunoblotting analysis with cropped blots of FOXO3A protein levels in teloHAEC treated with PE and PVC alone or in combination, or pretreated with iPCSK9 and then exposed to PE, PVC, and PE + PVC. M, molecular weight markers; lane 1, Ctr; lane 2, PE; lane 3, PVC; lane 4, PE + PVC; lane 5, iPCSK9; lane 6, PE + iPCSK9; lane 7, PVC + iPCSK9; lane 8, PE + PVC + iPCSK9. **B** Representative cropped blots with relative immunoblotting analysis of FOXO3A in EC transfected with NT or with siSIRT6 and then exposed to PE + PVC (NT or siSIRT6 + PE + PVC), to 100 µg/mL iPCSK9 (NT or siSIRT6 + iPCSK9), or pretreated with 100 µg/mL iPCSK9 for 8 h before exposure to PE + PVC (NT or siSIRT6 + PE + PVC + iPCSK9). M, molecular weight markers; lane 1, Ctr; lane 2, NT; lane 3, NT + PE + PVC; lane 4, NT + iPCSK9; lane 5, NT + PE + PVC + iPCSK9; lane 6, siSIRT6; lane 7, siSIRT6 + PE + PVC; lane 8, siSIRT6 + iPCSK9; lane 9, siSIRT6 + PE + PVC + iPCSK9. Data are expressed as mean ± SD of *n* = 3 experiments. **p < 0.01 versus Ctr; †*p* < 0.05 versus PE; ‡*p* < 0.05 versus PVC; + *p* < 0.05 versus PE + PVC; °*p* < 0.01 versus NT

To explain the association between SIRT6 and PCSK9, a common molecular target, FOXO3A [38], was investigated. To this end, the possible FOXO3A/SIRT6/PCSK9 axis in EC under MP stimulation was assessed. Immunoblotting analysis revealed that MP treatment led to downregulated FOXO3A expression levels (*p* < 0.01 versus Ctr), while iPCSK9 reverted this effect (*p* < 0.05 versus PE, *p* < 0.05 versus PVC, *p* < 0.05 versus PE + PVC) (Fig. 9A).

Moreover, SIRT6 silencing slightly decreased FOXO3A expression levels (*p* < 0.05 versus NT), and worsened FOXO3A downregulation in EC under PE + PVC treatment (siSIRT6 + PE + PVC) (*p* < 0.05 versus PE + PVC) (Fig. 9B).

Of note, in siSIRT6 EC, iPCSK9 pretreatment was unable to oppose the MP-induced FOXO3A downregulation. In contrast, compared with SIRT6-expressing cells treated with iPCSK9, in siSIRT6 + iPCSK9 EC a lower FOXO3A protein expression was observed (*p* < 0.05), supporting the involvement of SIRT6 in the action of iPCSK9 and indicating the involvement of the FOXO3A/SIRT6/PCSK9 network in endothelial redox function (Fig. 9B).

## Discussion

MP pollution has emerged as a critical environmental issue, raising significant concern about its impact on both ecosystems and human health.

This study provides, for the first time, significant insights into the in vitro deleterious effects of MPs on endothelial function and provides additional knowledge on the beneficial effects of PCSK9 inhibitors at the vascular level. Specifically, we demonstrated that exposure of EC to PE and PVC MPs induced excessive ROS production and mitochondrial metabolism impairment, increased inflammatory state, and promoted activation of autophagic pathway, cell cycle arrest, and apoptosis. At molecular levels, the deleterious effects of MPs correlated with increased PCSK9 levels and a downregulation of SIRT6 and FOXO3A protein levels. Of note, the use of iPCSK9 evolocumab ameliorated the deleterious effects induced by MPs, restoring the levels of SIRT6 and FOXO3A. Conversely, SIRT6 silencing removed the beneficial effects of iPCSK9 evolocumab, exacerbating inflammation and oxidative stress.

MPs can enter the human body through inhalation, ingestion of contaminated food and water, and dermal contact, causing multiple toxic effects on various human organs and systems [4, 6]. Through ingestion, MPs can accumulate in the digestive tract [12], thus promoting gut flora dysbiosis, intestinal inflammation, and altered function of intestinal epithelial cells [39–41]. The occurrence of MPs in the human respiratory system [10] caused cellular senescence by increasing inflammation and oxidative stress [42]. Recently, MPs have also been shown in the renal system [43], and exposure of human kidney cells to MPs determined an increase in ROS production, pro-inflammatory cytokine release, and cell death mechanisms [44, 45]. MPs can also cross the blood–brain barrier and accumulate in the central nervous system [13], leading to neurotoxicity and neurodegeneration associated with increased oxidative stress [46]. Furthermore, the presence of MPs has recently been confirmed in both the male and female reproductive systems and placenta, posing significant risks also to fetal development through hormonal disturbances, oxidative stress, and cellular damage [14, 16, 18, 47–51]. A recent high-impact study showed that MPs within atherosclerotic plaques exacerbated cardiovascular risk [21]. Of the 304 patients undergoing carotid endarterectomy who participated in that study, 257 completed follow-up. Patients with carotid plaques presenting micro–nanoplastics (MNPs), such as PE and PVC, had a higher risk of major cardiovascular events than patients without MNPs in atheroma [21]. A correlation was found between the amount of MNPs and increased levels of pro-inflammatory cytokines IL-18, IL-1β, IL-6, and TNF-α, as well as the markers CD3 and CD68, and decreased collagen content [21]. A subsequent study involving patients undergoing arterial or venous thrombectomy owing to ischemic stroke (IS), myocardial infarction (MI), or deep vein thrombosis (DVT) revealed the presence of MPs, including PE and PVC, in 80% of the samples, with a higher level of the hypercoagulability marker d-dimer in patients presenting MPs [52]. Therefore, MPs represent a new emerging risk factor for several disorders, including CVD.

The vascular endothelium plays a crucial role in maintaining vascular homeostasis [53], while dysfunctional endothelial cells represent a critical event in the pathogenesis of CVD, including atherosclerosis [23, 34, 54]. At the cellular level, MPs can translocate across gastrointestinal or respiratory barriers into the bloodstream [19] and then interact with EC lining blood vessels, leading to endothelial activation via increased expression of adhesion molecules and inflammatory cytokines, resulting in endothelial dysfunction and CVD progression [24, 25].

In accordance with previous in vitro and in vivo studies indicating the ability of MPs to exacerbate oxidative stress [55, 56], our results showed the ability of PE and PVC MPs to induce mitochondrial ROS accumulation and impairment of mitochondrial respiration, indicating significant disruption of mitochondrial function and cellular redox balance, a crucial determinant of EC health and longevity [57, 58]. A recent study showed that MPs, including PE and PVC, activated ROS signaling and altered the expression of mitochondria-related proteins in human lung epithelial cells, promoting mitochondrial dysfunction by disrupting redox homeostasis [42].

PVC-MPs also exacerbated ROS production in human blood lymphocytes and human intestinal epithelial cell lines, along with collapse of mitochondrial membrane potential (MMP) [59, 60].

Moreover, the observed upregulation of pro-inflammatory modulators MCP-1, VCAM1, and ICAM1 following MP exposure supports the known pro-inflammatory properties of these pollutants [57, 58].

The altered induction of autophagy and cell death mechanisms by MPs shown in our study corroborates the notion that MPs disrupt vascular homeostasis [27, 61]. A previous study by Lee et al. reported that exposure of EC to PS-MP (80 μg/mL) impaired angiogenesis through repression of vascular endothelial growth factor (VEGF) signaling, and affected cell viability through activation of autophagy and necrosis processes [27]. Autophagy, typically a protective response to cellular stress [61], becomes dysregulated and excessive under PE and PVC exposure, leading to increased cell death and contributing to endothelial damage and vascular complication. Although both PE and PVC MPs altered endothelial phenotype in our experimental model, no significant differences were observed between the two MPs in terms of inflammatory response, mitochondrial dysfunction, oxidative stress, and cell death. These findings suggest that the detrimental effects on endothelial function may represent a common cellular response to MP exposure. In agreement with this, previous studies reported that other MP polymers were able to impair endothelial and nonendothelial cell homeostasis by inducing oxidative stress, inflammation, and apoptosis, although the intensity of these effects may vary depending on particle size and surface properties [7]. Furthermore, in our experiments, the three endothelial cell types (immortalized endothelial human aorta cell line, teloHAEC, human umbilical vein endothelial cells, HUVEC, and human coronary artery endothelial cells, HCAEC) showed similar alterations following MP exposure. These data suggest that the harmful effects of MPs on endothelial function are not limited to a specific vascular district but rather represent a response with potential systemic implications for vascular pathology, including atherosclerotic plaque formation, thrombo-inflammatory processes, and ischemic heart disease [21, 52]. The consistency of these alterations therefore suggests that MP exposure may contribute to a common pathogenic mechanism underlying several vascular disorders.

These events were related to an increase in PCSK9 protein levels and downregulation of SIRT6 protein levels.

PCSK9 protein is a member of the proprotein convertase family, playing a key role in regulating lipid metabolism [62], and its overexpression is associated with increased risk of CVD, including the formation of atherosclerosis plaque [32, 63]. At the vascular level, PCSK9 has a direct and significant impact on EC, promoting pro-inflammatory pathways, oxidative stress, and cell death mechanisms, thus contributing to endothelial dysfunction and progression of CVD independently of cholesterol levels [28, 64, 65].

PCSK9 inhibitors, including evolocumab, are a class of lipid-lowering drugs effective in the treatment of CVD by reducing LDL-C levels [66].

Recent studies suggested a potential beneficial effect of iPCSK9 independent of LDL-C reduction [67], by improving endothelial function, reducing oxidative stress, inflammation, and cell death [68]. Moreover, iPCSK9 was also able to counteract IL-6-induced endothelial dysfunction and that the protective effects of iPCSK9 could be mediated, at least in part, by SIRT3 [28]. Furthermore, iPCSK9 protected the endothelium during sepsis via SIRT4 modulation, through anti-inflammatory and anti-autophagic mechanisms [69]. Accordingly, results from this study showed that iPCSK9 mitigated the MP-induced endothelial dysfunction, by counteracting the inflammatory response, mitochondrial impairment, autophagy mechanism, and apoptosis and restoring SIRT6 protein levels.

SIRT6 is known to be involved in the regulation of DNA repair, longevity and aging, energy metabolism, and inflammation, crucial pathways for the development of CVD [33, 70, 71]. At the endothelial level, SIRT6 contributed to vascular function and integrity, protecting EC from premature senescence and ROS accumulation through pathways that prevent endothelial dysfunction in hyperglycemic conditions, chronic inflammation, and atherosclerosis [31, 70–72]. The significant downregulation of SIRT6 observed in EC under MP treatment suggested that SIRT6 activity could be essential to protect EC, also from environmental insults. Indeed, in SIRT6-silenced EC, exacerbation of MP-induced deleterious effects occurred and the beneficial effects of iPCSK9 failed. MP-induced upregulation of PCSK9 may be mediated by redox-sensitive and inflammatory pathways, including nuclear factor kappa-light-chain-enhancer of activated B cells (NF-κB) activation, which are recognized regulators of PCSK9 transcription [28, 64, 65]. This is consistent with the marked oxidative stress and pro-inflammatory response observed in our experiments. In addition, the inverse relationship between PCSK9 and SIRT6 observed in EC exposed to PE and PVC suggests that PCSK9 may contribute to SIRT6 downregulation through mechanisms involving increased ROS production and persistent inflammatory signaling. Given that SIRT6 represents a critical regulator of DNA repair, redox balance, and endothelial integrity [33, 70, 71], its repression by PCSK9 may represent a key mechanism by which MPs impair endothelial homeostasis. The ability of evolocumab to restore SIRT6 expression further supports this hypothesis, indicating that the PCSK9–SIRT6 axis may represent a critical molecular link in the endothelial response to MP exposure. Nevertheless, additional studies will be required to dissect the precise molecular interactions connecting PCSK9 to SIRT6 repression. SIRT6 overexpression also protected against ischemia/reperfusion injury by increasing the nuclear translocation of FOXO3A and its ability to bind antioxidant gene promoters [73]. FOXO3A is a transcription factor implicated in the oxidative stress response, and in vascular aging and atherosclerosis by protecting EC via upregulation of catalase and superoxide dismutase 2 antioxidant enzymes [73, 74].

Our results reveal that MP exposure led to downregulation of the FOXO3A expression level in EC, while treatment with iPCSK9 opposed this effect. Meanwhile, SIRT6 silencing also resulted in a downregulation of FOXO3A expression and worsened the MP-induced decrease in FOXO3A levels, and the ability of iPCSK9 to mitigate this phenotype was impaired. The modulation of FOXO3A suggests a complex regulatory network involving SIRT6, PCSK9, and FOXO3A in endothelial health. In the liver, FOXO3A cooperates with SIRT6 to suppress PCSK9 expression by increasing recruitment of SIRT6 to PCSK9 promoter, thus leading to histone deacetylation and downregulation of PCSK9 transcription [38]. This regulatory mechanism has been reported in the maintenance of low LDL-C levels, thereby reducing the risk of CVD [38]. We speculated that a similar molecular mechanism could occur in the vascular EC. Undoubtedly, further in vitro and in vivo studies are necessary to provide and clarify the FOXO3A/SIRT6/PCSK9 axis in EC under MP treatment.

Overall, this study showed, for the first time, the harmful effects of PE and PVC on vascular endothelium and demonstrated that inhibition of PCSK9 by evolocumab provides a protective mechanism against MP-induced endothelial dysfunction through modulation of SIRT6 and FOXO3A pathways. It is important to highlight that the MP concentrations used in the present in vitro study (70 µg/mL) are higher than those currently detected in human plasma and tissues. However, these concentrations were selected to simulate the cumulative cellular impact of chronic environmental exposure within a limited experimental timeframe. MPs, including PE and PVC, have been identified in various human biological compartments such as blood, placenta, lungs, and liver. In human blood, MP concentrations of up to 1.6 µg/mL have been observed, with substantial accumulation also observed in placental and hepatic tissues [75]. A median of 18 MPs/g has also been described in placental samples, with PE and PVC among the most frequently detected polymers [76]. Leonard et al. further reported polyethylene concentrations reaching 4.65 µg/mL in blood samples using micro Fourier-transform infrared (µFTIR)-based analysis [20]. Therefore, although the direct extrapolation of our findings to physiological exposure is limited, the experimental model offers valuable mechanistic insights into the potential long-term vascular effects of MPs. Further research is needed to highlight the relevance of these findings in vivo and explore the benefits and potential clinical applications of PCSK9 inhibitors in mitigating environmental pollutant-induced cardiovascular risks.

## Conclusions

The present study provides evidence of the adverse effects of PE and PVC MPs on endothelial function, suggesting that MPs represent a significant environmental threat to vascular health. The demonstrated efficiency of PCSK9 inhibitors in mitigating these effects by modulating the SIRT6/FOXO3A axis open the way for further in vivo studies on the role of iPCSK9 as a promising therapeutic strategy to reduce cardiovascular risk associated with plastic pollution exposure.

## Supplementary Information

Supplementary material 1.

## Data Availability

The datasets used and analyzed during the current study are available from the corresponding author on reasonable request.

## Abbreviations

MPs: Microplastics
TeloHAEC: Human aortic endothelial cells
HUVEC: Human umbilical vein endothelial cells
HCAEC: Human coronary artery endothelial cells
PE: Polyethylene
PVC: Polyvinyl chloride
PCSK9: Proprotein convertase subtilisin/kexin type 9
iPCSK9: Proprotein convertase subtilisin/kexin type 9 inhibitor
IL-18: Interleukin-18
IL-1 β: Interleukin-1β
IL-6: Interleukin-6
TNF-α: Tumor necrosis factor-α
CVD: Cardiovascular diseases
EC: Endothelial cells
PS-MPs: Polystyrene microplastics
SIRT: Sirtuins
HBSS: Hanks’ balanced salt solution
Ctr: Control cells
CI: Combination index
GAPDH: Glyceraldehyde 3-phosphate dehydrogenase
PI: Propidium iodide
OCR: Oxygen consumption rate
FCCP: Carbonyl cyanide-4(trifluoromethoxy) phenylhydrazone
siRNA: Small interfering RNA
SDS-PAGE: Sodium dodecyl sulfate–polyacrylamide gel electrophoresis
SIRT6: Sirtuin 6
p62: Anti-sequestosome-1
ATG5: Anti-autophagy related 5
FOXO3A: Anti-Forkhead Box O3
ROS: Reactive oxygen species
MNPs: Micro–nanoplastics
IS: Ischemic stroke
MI: Myocardial infarction
DVT: Deep vein thrombosis
MMP: Mitochondrial membrane potential
VEGF: Vascular endothelial growth factor
