# Supplementary material for: PCSK9 inhibition ameliorates microplastic-induced endothelial redox imbalance via SIRT6 modulation
Source: Cell Mol Biol Lett. 2025 Dec 22;31:9. doi: 10.1186/s11658-025-00838-z (PMC12838487; doi:10.1186/s11658-025-00838-z)
Supplement: Supplementary file 1 — Supplementary material 1. [file 11658_2025_838_MOESM1_ESM.docx]

**PCSK9 inhibition ameliorates microplastics-induced endothelial redox imbalance via SIRT6 modulation**

Nunzia D’Onofrio^1, 5¶^_,_ Isabella Donisi^1¶^, Vitale Del Vecchio^2^, Francesco Prattichizzo^3^, Valeria Pellegrini^3^, Michelangela Barbieri^4,5^, Antonio Ceriello^3^, Raffaele Marfella^4,5^, Giuseppe Paolisso^4,5#^, Maria Luisa Balestrieri^1,5#*^

^a^Department of Precision Medicine, University of Campania Luigi Vanvitelli, Via L. De Crecchio 7, 80138 Naples, Italy; nunzia.donofrio@unicampania.it (ND); isabella.donisi@unicampania.it (ID); marialuisa.balestrieri@unicampania.it (MLB).

^b^ Department of Experimental Medicine, University of Campania Luigi Vanvitelli, Via Luciano Armanni 5, 80138 Naples, Italy; vitale.delvecchio@unicampania.it (VDV).

^c^ IRCCS MultiMedica, Via Fantoli 16/15, 20138, Milan, Italy; francesco.prattichizzo@multimedica.it (FP); valeria.pellegrini@multimedica.it (VP); antonio.ceriello@hotmail.it (AC)

^d^ Department of Advanced Clinical and Surgical Sciences, University of Campania Luigi Vanvitelli, Piazza Miraglia, 80138, Naples, Italy; michelangela.barbieri@unicampania.it (MB); raffaele.marfella@unicampania.it (RM); giuseppe.paolisso@unicampania.it (GP)

^e^ Research Center for Environmental Pollution and Cardiovascular Diseases, University of Campania Luigi Vanvitelli, Piazza Miraglia, 80138, Naples, Italy.

^¶^ Authors shared first authorship

^#^ Authors contributed equally to this work and share last authorship

*Correspondence: [marialuisa.balestrieri@unicampania.it](mailto:marialuisa.balestrieri@unicampania.it); Tel.: +39-081-5665865

SUPPLEMENTARY MATERIALS


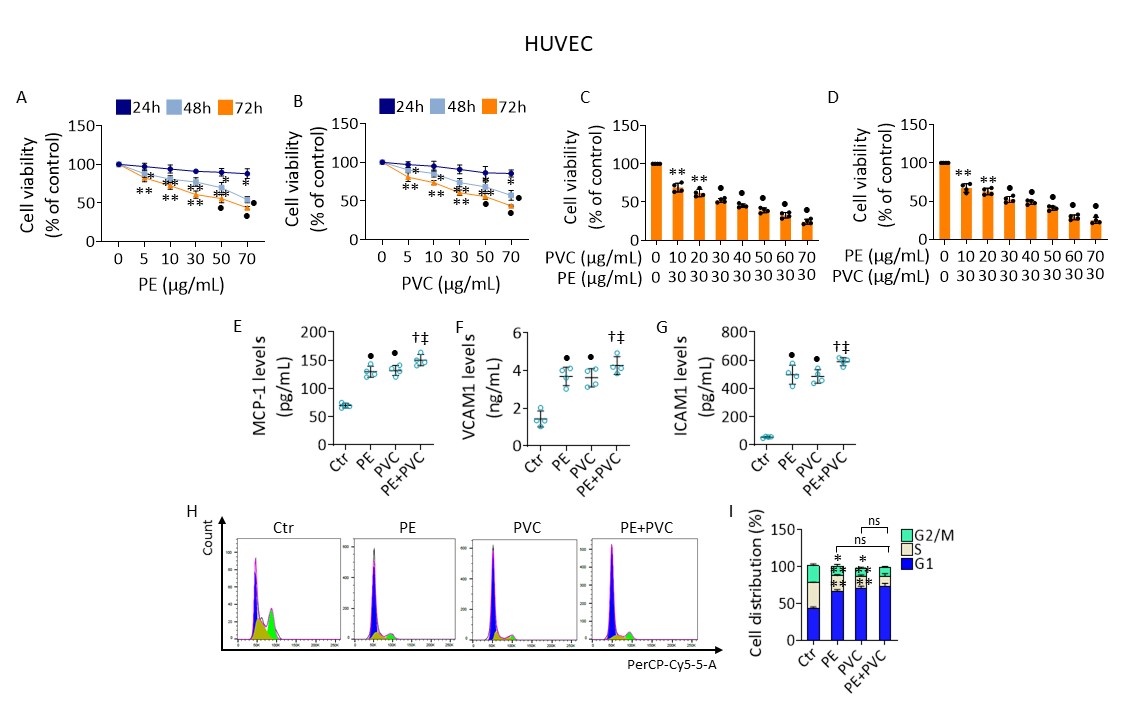


**Supplementary Figure S1. *MPs effects on HUVEC*.** Cell viability evaluated in HUVEC exposed to different concentrations of (A) PE and (B) PVC (0-70 µg/mL) for 24, 48 and 72 h. (C-D) Cell viability assessed after 48 h of treatment with PE or PVC (30 µg/mL) and increasing concentration of PVC or PE (0-70 µg/mL), respectively. Evaluation of (E) MCP-1, (F) VCAM1 and (G) ICAM1 content in HUVEC treated with PE (70 µg/mL), PVC (70 µg/mL) or PE+PVC (30 µg/mL+30 µg/mL) for 48 h. (H-I) Representative cell cycle detection by FACS analysis in EC exposed to MPs. Data are expressed as the mean ± SD of *n* = 4 independent experiments. * *p* < 0.05 *vs* 0 µg/mL or Ctr; ** *p* < 0.01 *vs* 0 µg/mL or Ctr; • *p* < 0.001 *vs* 0 µg/mL or Ctr; † *p* < 0.05 *vs* PE; ‡ *p* < 0.05 *vs* PVC; ns, not significant *vs* PE and PVC.


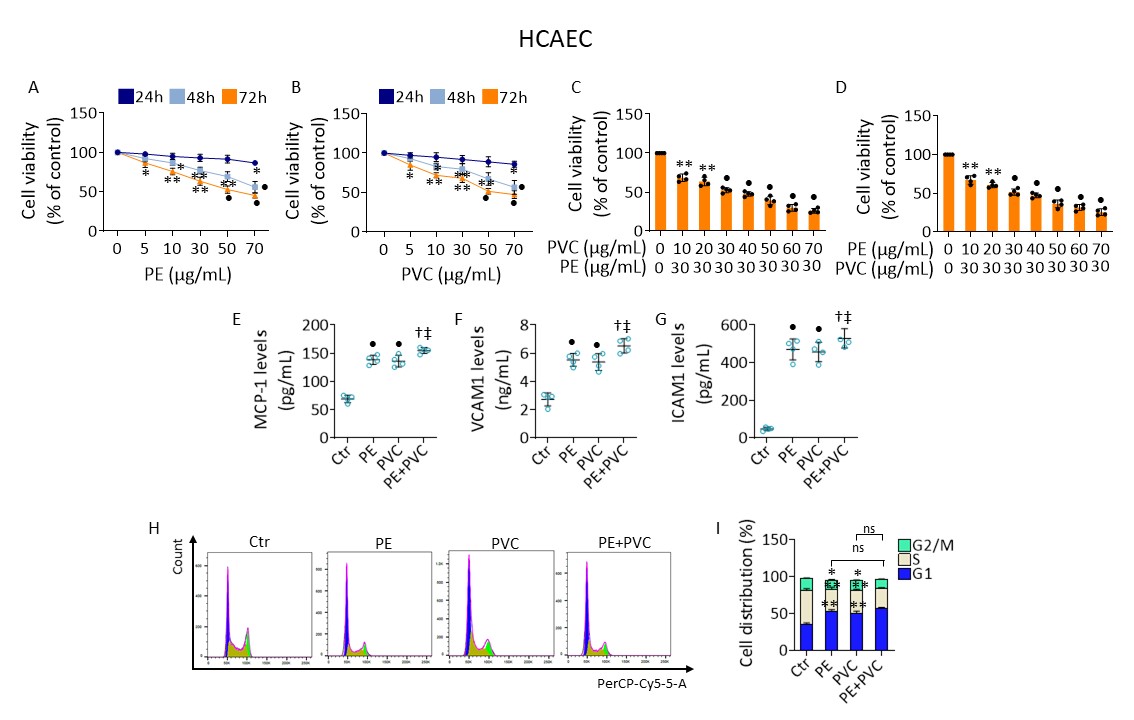


**Supplementary Figure S2. *MPs effects on HCAEC*.** Cell viability evaluated in HCAEC exposed to different concentrations of (A) PE and (B) PVC (0-70 µg/mL) for 24, 48 and 72 h. (C-D) Cell viability assessed after 48 h of treatment with PE or PVC (30 µg/mL) and increasing concentration of PVC or PE (0-70 µg/mL), respectively. Evaluation of (E) MCP-1, (F) VCAM1 and (G) ICAM1 content in HCAEC treated with PE (70 µg/mL), PVC (70 µg/mL) or PE+PVC (30 µg/mL+30 µg/mL) for 48 h. (H-I) Representative cell cycle detection by FACS analysis in EC exposed to MPs. Data are expressed as the mean ± SD of *n* = 4 independent experiments. * *p* < 0.05 *vs* 0 µg/mL or Ctr; ** *p* < 0.01 *vs* 0 µg/mL or Ctr; • *p* < 0.001 *vs* 0 µg/mL or Ctr; † *p* < 0.05 *vs* PE; ‡ *p* < 0.05 *vs* PVC; ns, not significant *vs* PE and PVC.


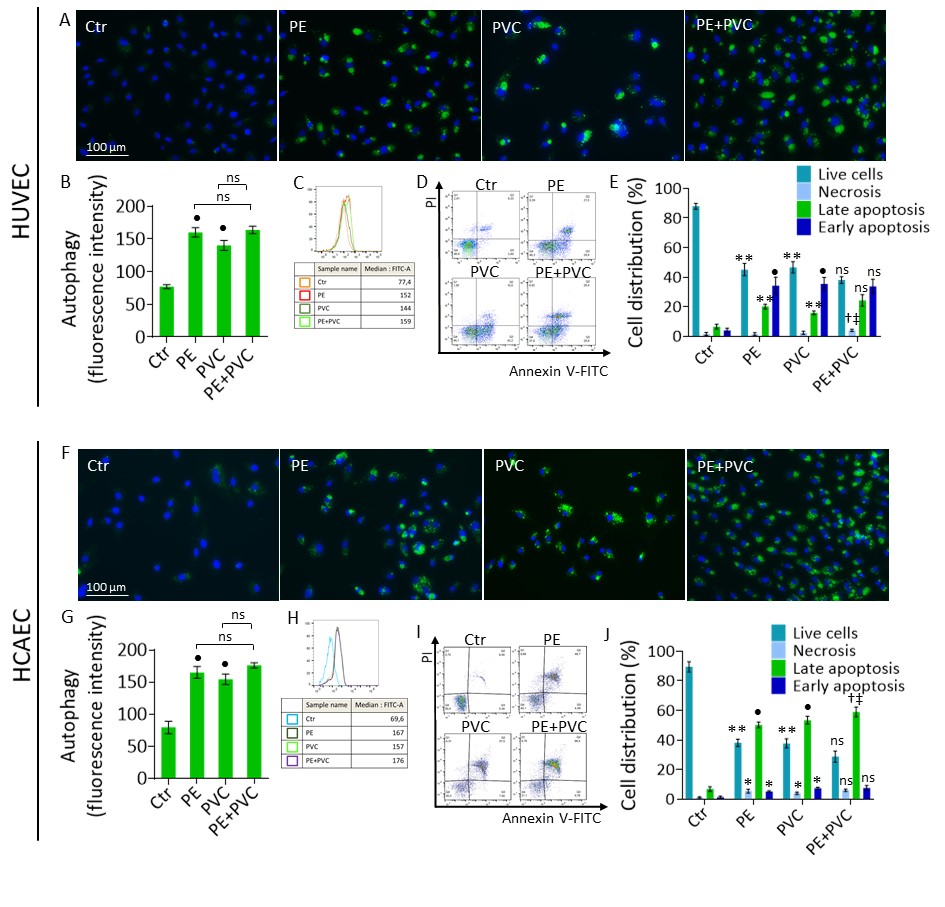


**Supplementary Figure S3. *MPs triggered cell death on EC*.** Representative fluorescent images and FACS analysis of autophagy detection and representative dot plots and analysis of annexin V-FITC and PI-staining in (A-E) HUVEC and (F-J) HCAEC treated for 48 h with PE (70 µg/mL), PVC (70 µg/mL) or combined PE+PVC (30 µg/mL+30 µg/mL). Results are expressed as median fluorescence intensity (MFI). Data are expressed as mean ± SD of *n* = 3 experiments. Q1: necrotic cells; Q2: late apoptotic cells; Q3: early apoptotic cells; Q4: viable cells. Scale bars = 100 μm. * *p*< 0.05 *vs* Ctr; ** *p* < 0.01 *vs* Ctr, • *p* < 0.001 *vs* Ctr; ns, not significant *vs* PE and PVC.


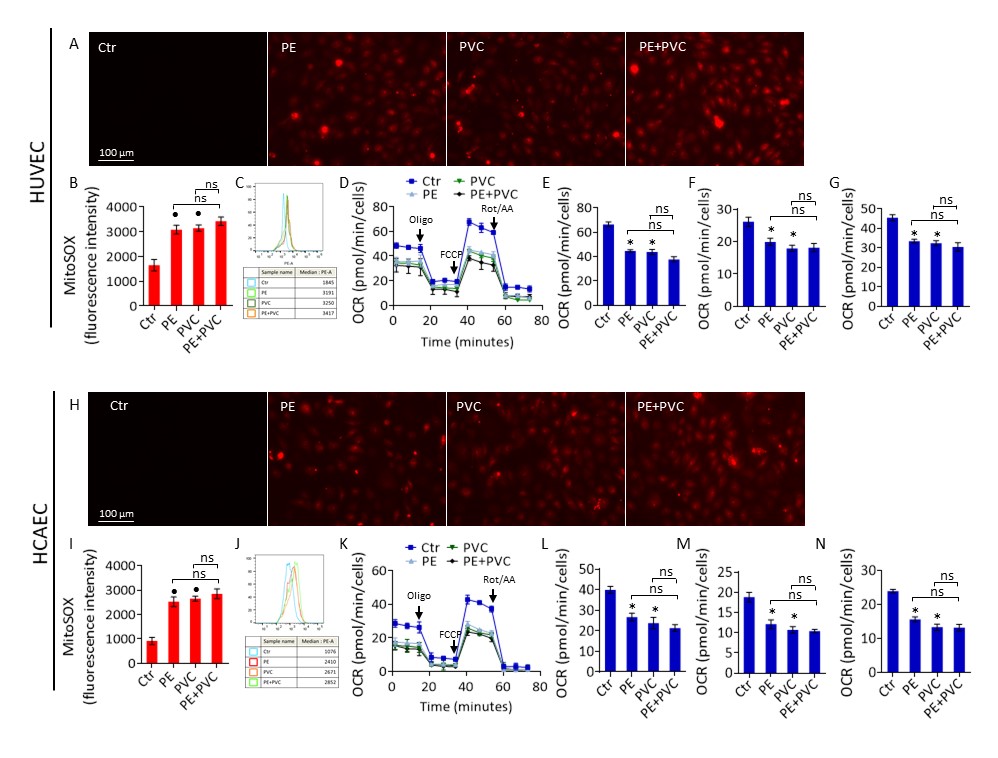


**Supplementary Figure S4. *MPs induced mitochondrial alteration in EC.*** Representative fluorescent images and FACS analysis of mitochondrial ROS levels and mitochondrial analysis of oxygen consumption rate, maximal respiration, ATP production and basal respiration measured in (A-G) HUVEC and (H-N) HCAEC treated with treated for 48 h with PE (70 µg/mL), PVC (70 µg/mL) or combining PE (30 µg/mL) and PVC (30 µg/mL). Results are expressed as median fluorescence intensity (MFI). Data are expressed as mean ± SD of *n* = 3 experiments. Scale bars = 100 μm. * *p* < 0.05 *vs* Ctr; • *p* < 0.001 *vs* Ctr; ns, not significant *vs* PE and PVC.


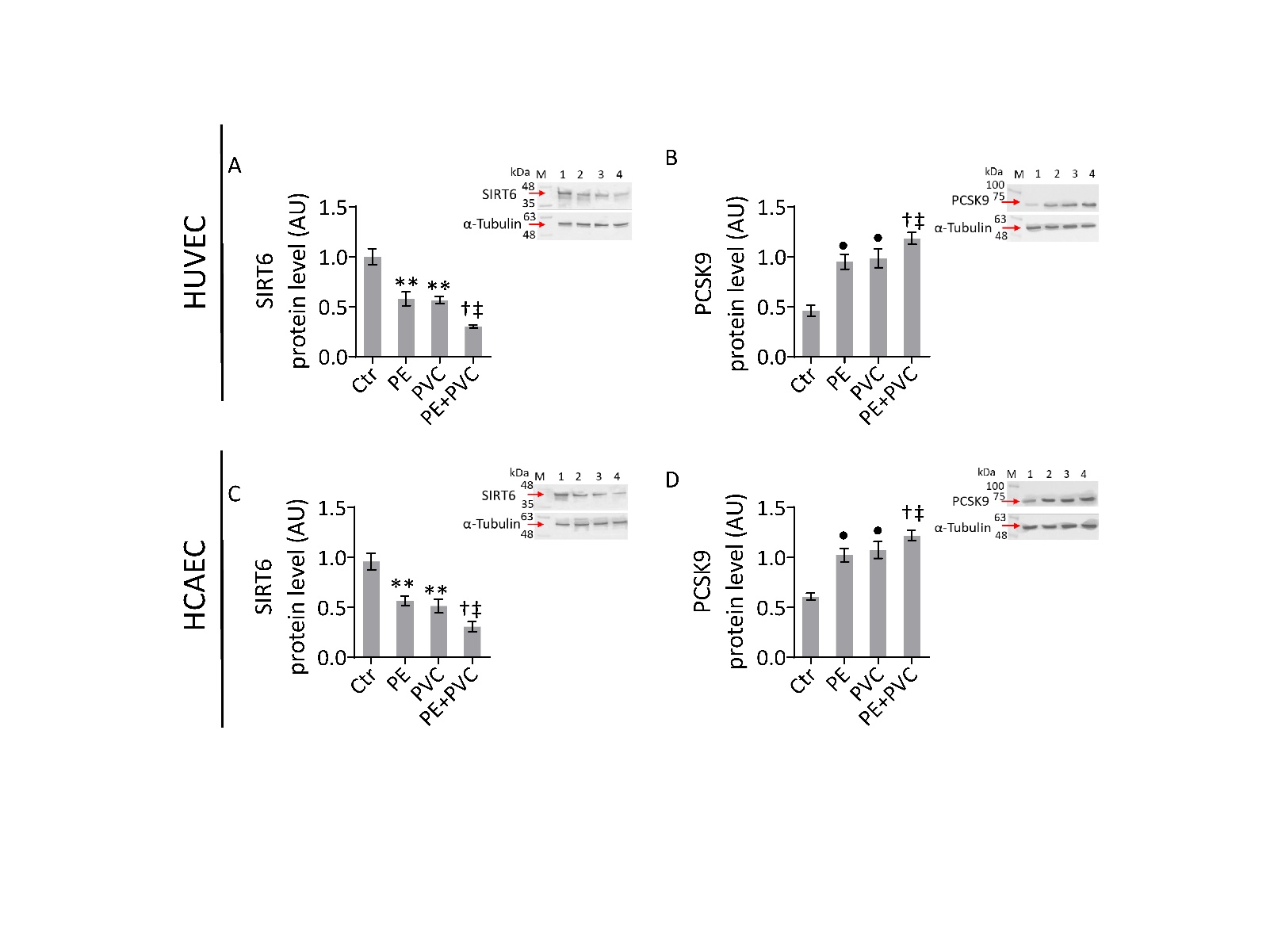


**Supplementary Figure S5. *MPs modulated the protein expression levels of SIRT6 and PCSK9 in EC.*** Immunoblotting analysis of SIRT6 and PCSK9 protein levels in (A,B) HUVEC and (C,D) HCAEC exposed to PE (70 µg/mL), PVC (70 µg/mL) or PE+PVC (30 µg/mL+30 µg/mL) for 48 h. Data are expressed as mean ± SD of *n* = 3 experiments. M, molecular weight markers; lane 1, Ctr; lane 2, PE; lane 3, PVC; lane 4, PE+PVC. ** *p* < 0.01 *vs* Ctr; • *p* < 0.001 *vs* Ctr; † *p* < 0.05 *vs* PE; ‡ *p* < 0.05 *vs* PVC.


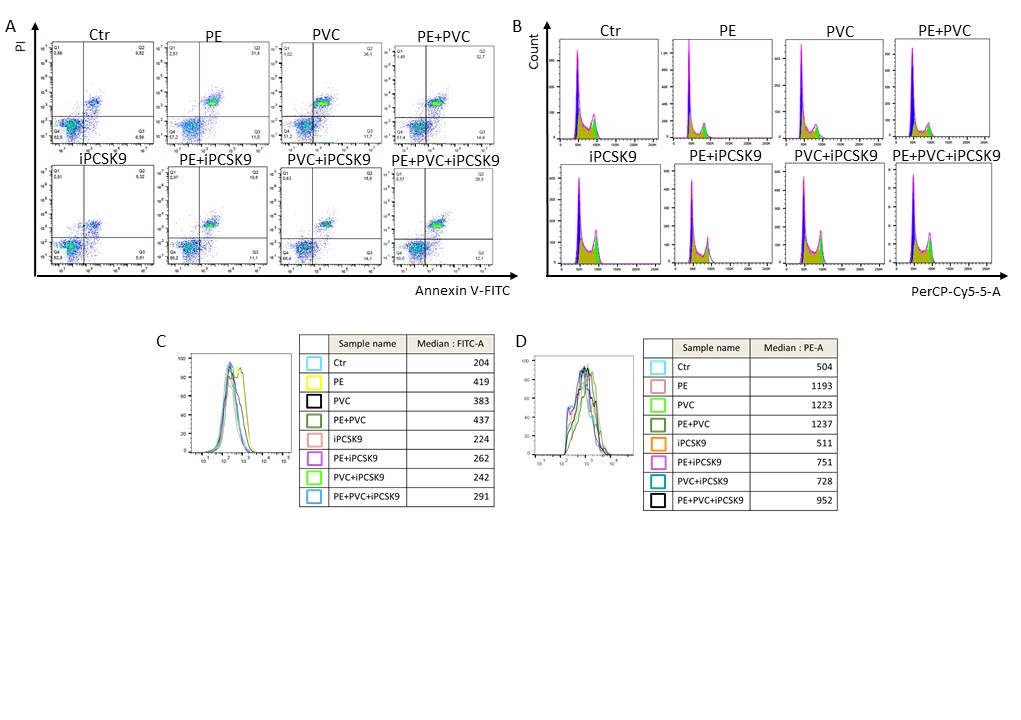


**Supplementary Figure S6. *FACS analyses.*** FACS analyses of (A) annexin V-FITC and PI-staining, (B) cell cycle (C) autophagy and (D) mitochondrial ROS levels performed on teloHAEC treated for 48 h with PE and PVC alone or in combination PE+PVC, or pre-treated with iPCSK9 and then exposed to PE, PVC or PE+PVC. Q1: necrotic cells; Q2: late apoptotic cells; Q3: early apoptotic cells; Q4: viable cells.
